# Mapping the effects of exercise on cognition and physical function: a scoping review of rodent studies

**DOI:** 10.3389/fphys.2026.1769763

**Published:** 2026-04-15

**Authors:** Muhammad Hafiz Zuhdi Fairof, Norwahidah Abdul Karim, Theng Choon Ooi, Leong Chen Lew, Arimi Fitri Mat Ludin, Nor Fadilah Rajab

**Affiliations:** 1 Centre for Healthy Ageing and Wellness HCARE, Faculty of Health Sciences, Universiti Kebangsaan Malaysia, Kuala Lumpur, Federal Territory of Kuala Lumpur, Malaysia; 2 Programme of Biomedical Science, Faculty of Health Sciences, Universiti Kebangsaan Malaysia, Kuala Lumpur, Federal Territory of Kuala Lumpur, Malaysia; 3 Department of Biochemistry, Faculty of Medicine, Universiti Kebangsaan Malaysia, Bandar Tun Razak, Cheras, Federal Territory of Kuala Lumpur, Malaysia; 4 Premier Integrated Labs Sdn. Bhd, Pandan Indah, Kuala Lumpur, Selangor, Malaysia

**Keywords:** brain-muscle crosstalk, exercise, rodent, scoping review, timing

## Abstract

**Introduction:**

Exercise, including both resistance and aerobic modalities, is widely recognised for its cognitive and physical health benefits, such as enhanced cognitive performance, improved physical fitness, and disease prevention. An emerging field, known as chrono-exercise, examines how the timing of exercise interacts with circadian rhythms to maximise these benefits. With ageing, cognitive decline and physical impairment become increasingly prevalent, contributing to a higher risk of age-related disorders. This scoping review aims to explore whether strategies, particularly those involving brain–muscle crosstalk, can mitigate these declines.

**Method:**

This review was conducted in accordance with PRISMA (Preferred Reporting Items for Systematic Reviews and Meta-Analyses) guidelines. Rodent studies were identified from PubMed, Scopus, Cochrane, and ProQuest databases. Included studies examined the effects of various exercise interventions, including aerobic and resistance training, on neurobehavioural outcomes, body composition, and physiological and biochemical regulatory mechanisms.

**Result:**

Overall, exercise interventions demonstrate beneficial effects on cognitive and physical health. These effects appear to be enhanced when exercise is aligned with circadian rhythms and appropriately supplemented, highlighting their potential as non-invasive strategies to mitigate age-related decline.

**Conclusion:**

These findings emphasise the need for further research to optimise exercise protocols and develop personalised interventions aimed at improving cognitive and physical health in ageing populations.

## 1 Introduction

Physical exercise is widely recognised as a key determinant of health, exerting beneficial effects on physical performance, cognitive function, and mental well-being. Aerobic and resistance exercise improve cardiovascular fitness, metabolic regulation, and musculoskeletal integrity, while also reducing the risk of chronic conditions such as diabetes, cardiovascular disease, and certain cancers (Gomez-Pinilla and Hillman, 2013; Hawley et al., 2014; Hargreaves and Spriet, 2020). Beyond these systemic effects, accumulating evidence indicates that exercise may support brain health by promoting neuroplasticity, modulating oxidative stress and neuroinflammation, and improving mood and cognitive/emotional resilience (Camandola and Mattson, 2017; Consorti et al., 2021; Rai and Demontis, 2022).

The incorporated framework underlying these diverse benefits is brain–muscle crosstalk, which describes the bidirectional communication between skeletal muscle and the central nervous system via neural, endocrine, and metabolic pathways. Contracting muscles release myokines such as irisin and cathepsin B that influence synaptic plasticity, neurogenesis, and cognition, partly through brain-derived neurotrophic factor (BDNF) signalling (Pedersen et al., 2019). Similarly, the central motor regulates muscle activity and adaptation, refining voluntary and involuntary movement (He et al., 2018). This mutual signalling provides a mechanistic basis for how exercise can simultaneously influence physiological performance, cognitive function, and emotional regulation.

At the physiological level, aerobic and resistance training provide distinct yet complementary adaptations. Resistance exercise promotes muscle hypertrophy through increased protein synthesis (McGlory et al., 2017), whereas aerobic exercise enhances cardiovascular efficiency, angiogenesis, and oxygen delivery (Nystoriak and Bhatnagar, 2018). These structural changes are accompanied by biochemical adaptations, including improved mitochondrial function, glucose homeostasis, and hormonal regulation, which collectively support metabolic health and stress resilience (Hackney and Lane, 2015; Sellami et al., 2019). Importantly, such peripheral adaptations potentially feed back to the brain by enhancing cerebral blood flow, synaptic plasticity, and neurogenesis, thereby influencing learning, memory, and affective processes (Gomez-Pinilla and Hillman, 2013; Di Liegro et al., 2019; Ren and Xiao, 2023).

Despite this growing evidence, current research remains fragmented. Many studies focus on isolated outcomes such as muscle hypertrophy, cardiovascular fitness, or cognitive performance without integrating these findings within an incorporated muscle–brain framework. Moreover, exercise interventions differ widely in modalities, limiting cross-study comparability. As a result, it remains unclear how different exercise modalities concurrently shape structural and body composition, neurobehavior, physiological function and biochemical regulation and which mechanisms consistently link physical and cognitive adaptations.

An additional, emerging concept is chrono-exercise, which observes how circadian rhythms influence responses to physical exercise. Biological clocks regulate daily fluctuations in metabolism, hormone secretion, and neural activity (Kim et al., 2023). Evidence suggests that physical exercise and metabolic efficiency vary by time of day, with strength and oxidative capacity showing circadian modulation (Drust et al., 2005; van Moorsel et al., 2016; Gabriel and Zierath, 2019). However, the extent to which exercise timing modulates muscle–brain communication and cognitive outcomes remains insufficiently synthesised. Existing findings are dispersed across chronobiology, structural and body composition, neurobehavior, physiological function and biochemical regulation, with limited integrative analyses that connect circadian regulation to both physical and cognitive adaptations.

The inclusion of chrono-exercise in this review serves as an integrative framework rather than a prescriptive model. Although current evidence remains limited and heterogeneous, comparative findings suggest that exercise performed at different circadian phases elicits distinct neuroendocrine, inflammatory, metabolic and sleep-related responses. Morning exercise coinciding with cortisol awakening response (CAR) peak, transiently hyperactivates the HPA axis, elevating sympathetic tone and anabolic signalling elevate mitochondrial biogenesis (Yokokawa et al., 2018), yet risks prolonged glucocorticoid exposure, oxidative stress and blunted fat metabolism (Iwayama et al., 2015). Evening exercise aligning with circadian peaks in core temperature, neuromuscular power and melatonin onset optimises myokine release, cardiometabolic flexibility and mood elevation (Kim et al., 2023; Leota et al., 2025), but in the very late evening, it can provoke sleep disruptions via hyperarousal and delayed REM onset. Despite that, cognitive and molecular outcomes remain inconsistently reported. Importantly, substantial variability in study design, chronotype stratification and intensity control limits direct cross-study comparisons. Thus, the scientific value of integrating chrono-exercise lies in identifying mechanistic plausibility and research gaps rather than establishing optimal timing protocols.

Together, these limitations highlight a critical need for a structured synthesis that integrates exercise-induced adaptations across those domains. Accordingly, this scoping review aims to systematically map the existing literature on exercise interventions that affect structural and body composition, neurobehavior, physiological function, and biochemical regulation, with particular emphasis on muscle–brain crosstalk and the emerging role of exercise timing. By synthesising evidence across four selected research domains, this scoping review seeks to identify conceptual gaps, methodological heterogeneity and underexplored mechanisms, thereby providing a clear framework to guide future mechanistic and translational research.

## 2 Method

### 2.1 Review protocols

This scoping review critically examines studies in rodent models that explore how exercise influences both cognitive and muscle function through the framework of muscle–brain crosstalk, with particular emphasis on the type and timing of exercise interventions. While earlier reviews have documented the benefits of exercise, few have systematically considered how different exercise protocols, rodent strains, and life stages interact to shape outcomes. The novelty of this scoping review lies in two areas. First, it synthesises findings across diverse experimental designs to clarify how exercise interventions, including those applied at different time points, might affect the mechanisms underlying muscle–brain crosstalk. Second, it integrates results across multiple levels of evidence, such as behavioural, structural, physiological and biochemical, offering a more comprehensive and mechanistic perspective than previous reviews.

Three guiding questions frame this scoping review, (1) What exercise protocols and rodent models have been used to study muscle–brain crosstalk? (2) What are the effects of different exercise interventions on behavioural, anatomical, physiological and biochemical outcomes? and (3) What knowledge gaps remain regarding the role of exercise in shaping muscle–brain crosstalk across these levels?

To ensure a systematic approach, inclusion criteria were defined using the PCC framework instead. The Population consisted of rodent models, enabling mechanistic insights into muscle–brain communication. The Concept centred on the impact of exercise interventions, particularly timing and protocol, on both brain and muscle outcomes. The Context situated these findings across multiple domains, underscoring the novelty of this scoping review in synthesising timing- and protocol-specific effects within the broader framework of muscle–brain crosstalk.

The scoping review was conducted in line with PRISMA-P guidelines (Arksey and O'Malley, 2005) and adapted from the framework proposed by Levac, Colquhoun and O’Brien, with a specific focus on published animal studies. This approach helps overcome challenges in human research, such as small sample sizes and the reduced complexity of cell-based experiments. By concentrating on rodent models, this scoping review systematically evaluates how exercise interventions affect cognition and muscle health, while identifying gaps in understanding the mechanisms that link muscle and brain. No ethics approval was required for this scoping review.

The scoping review protocol followed six stages: (1) defining the study question, (2) identifying relevant studies, (3) selecting studies for inclusion, (4) extracting and charting data, (5) collating and summarising results, and (6) consulting with stakeholders. The protocol was registered on the Open Science Framework (https://doi.org/10.17605/OSF.IO/7XBGN).

A comprehensive search was conducted across PubMed, SCOPUS, the Cochrane Library and ProQuest, covering studies published from January 2012 to August 2023. Keywords such as “time of day,” “exercise,” and “rodent” were used to identify relevant literature. To ensure currency, the search was updated to include studies published between August 2023 and June 2025. The detailed search string strategy is presented in **Table 1**.

**Table 1:** Search string strategy.

Search string 1	"time of day" OR morning OR afternoon OR evening OR night OR timing OR "timing of exercise" OR biorhythm OR diurnal OR "diurnal timing" OR "circadian rhythm" OR "twenty four hour rhythm" OR "biological clock" OR "biological rhythm" OR "24-h cycle"
Search string 2	" exercise OR resistance training" OR training OR "physical activity" OR "aerobic training" OR "endurance training" OR "interval training" OR "high intensity interval training" OR "weight training" OR "moderate intensity continuous training"
Search string 3	rodent OR rat OR mouse OR mice
Search string 4	(("time of day" OR morning OR afternoon OR evening OR night OR timing OR "timing of exercise" OR biorhythm OR diurnal OR "diurnal timing" OR "circadian rhythm" OR "twenty four hour rhythm" OR "biological clock" OR "biological rhythm" OR "24-h cycle") AND ( exercise OR "resistance training" OR training OR "physical activity" OR "aerobic training" OR "endurance training" OR "interval training" OR "high intensity interval training" OR "weight training" OR "moderate intensity continuous training")) AND (rodent OR rat OR mouse OR mice)
Cochrane: Search string 1	"time of day" OR morning OR afternoon OR evening OR night OR timing OR "timing of exercise" OR biorhythm OR diurnal OR "diurnal timing" OR "circadian rhythm" OR "twenty four hour rhythm" OR "biological clock" OR "biological rhythm" OR "24 hours cycle"

### 2.2 Selection criteria

The selection criteria targeted studies investigating the effects of exercise on neurobehavioral, structural, physiological and biochemical regulation, specifically through brain-muscle cross-talk. Eligible studies included randomised controlled trials and primary studies published in English. Grey literature sources were not included due to feasibility constraints, and the language restriction was applied due to resource constraints, including the lack of translation capacity within the review team. Additionally, Non-rodent studies, drug-induced models, specific diet interventions and studies focused on psychologically induced disorders such as anxiety and depression were also excluded.

Two reviewers independently evaluated the titles and abstracts during the screening process, followed by a comprehensive assessment to match the selected studies with the scoping review objectives. Microsoft Excel was utilised for data extraction, which included study details such as author(s), publication year, study objectives, animal models, exercise protocols, control groups, intervention duration and effects on neurobehavioral, structural, physiological and biochemical regulation.

Findings that did not fit the chart were recorded separately as “noteworthy findings.” Before data extraction, the chart was reviewed in a meeting to ensure accuracy. Two researchers retrieved and documented data from the chosen publications separately, whereas other experts verified the accuracy of the information and settled differences through regular discussions. The team then summarised and analysed the information obtained, offering an overview of key study elements and summarising characteristics based on exercise type.

## 3 Result

**Figure 1:**
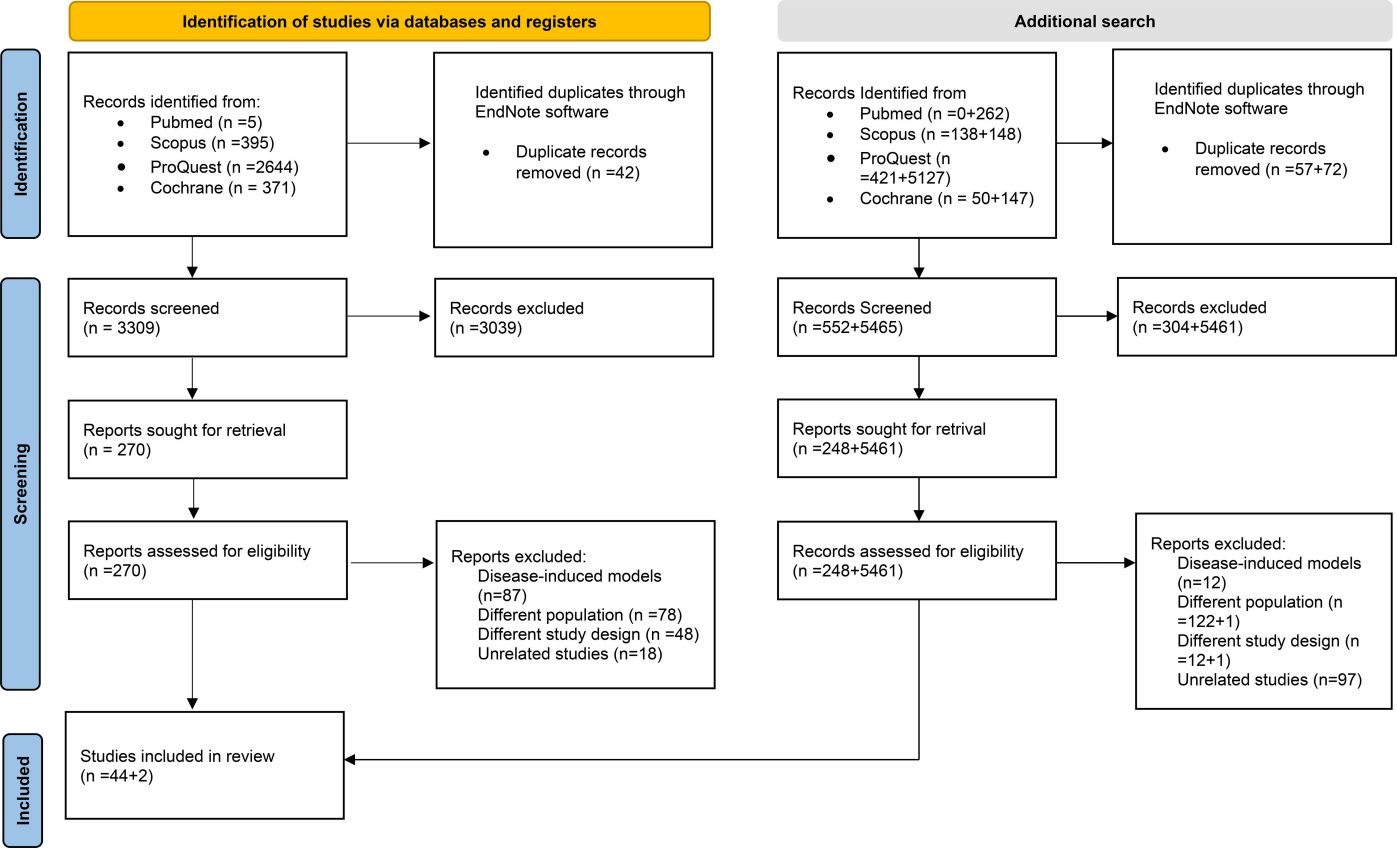
PRISMA flow diagram for the studies’ selection process.

The initial search across the four databases yielded 3,415 studies. After 42 duplicates were removed, 3,039 studies were excluded based on title and abstract screening. Full-text retrieval was performed for 270 studies, of which 231 were excluded for various reasons, including transgenic or disease-induced models (n=87), nonrodent species (n=78), unsuitable study designs (n=48) and unrelated topics (n=18). A total of 39 studies were initially included. After the search was updated, five additional studies met the inclusion criteria, resulting in a total of forty-four studies (n=44). **Figure 1** summarises those processes. Following the second search update, two additional studies were added, leading to a total of forty-six studies (n=46) included in this scoping review. This flow diagram represents the systematic review’s identification, screening, eligibility and inclusion steps.

Forty-six (n=46) of the included studies focused on aerobic exercise (n=41), four on resistance exercise (n=4), and one on both (n=1). Protocols for aerobic exercise included involuntary treadmill running (n=14), voluntary running wheels (n=18), swimming (n=8) and jumping. For resistance exercise, ladder climbing (n=2), downhill running (n=1) and running wheels with resistance (n=1) were used. **Table 2** presents the characteristics of the studies included. **Table 3** summarises the exercise protocol of the included studies. Forced exercise was the protocolized exercise, while voluntary exercise is free.

**Table 2:** Characteristics of included studies.

Author (Year)	Setting	Species	N	Age	Age Classification (Young < 4.5 months, 18 week, Old > 4.5 months, 18 months	Sex	Weight	Study Design	Control	Intervention
Guo et al., 2012	Standard	C57BL/6J	Not specified	28 months	Old	Not specified	Not specified	Experimental	Untreated testosterone	Treadmill running
Ghanbari-Niaki and Rahmati-Ahmadabad, 2013	Standard	Wistar	20	6–8 weeks	Young	Female	125–135 g	Experimental	Untrained	Treadmill running
Monaco et al., 2015	Standard	Sprague-Dawley	Not specified	25 weeks	Old	Not specified	~300 g	Experimental	Not specified	Treadmill running
Lalanza et al., 2015	Standard	Sprague-Dawley	66	12–14 weeks	Young	Male and female	Male: 517–550 g; Female: 312–330 g	Experimental	Sedentary, Control	Treadmill running
Hortemo et al., 2016	Standard	Sprague-Dawlesy	16	Not specified	Not specified	Male	~300 g	Experimental	Sedentary	Treadmill running
Li et al., 2016	Standard	Sprague-Dawley	16	18 months	Old	Male	240–340 g	Experimental	Sedentary	Treadmill running
Curzi et al., 2016	Standard	Sprague-Dawley	9	Not specified	Not specified	Male	200–300 g	Experimental	Sedentary	Treadmill running
Dalise et al., 2017	Standard	Sprague-Dawley	40	12 weeks	Young	Male	260 ± 15 g	Experimental	Sedentary	Treadmill running
Moshtagh et al., 2018	Standard	Wistar	Not specified	Not specified	Not Specified	Not specified	Not specified	Experimental	Not specified	Treadmill running
Aguiar et al., 2018	Standard	C57BL/6J	80	8 weeks	Young	Male	280–350 g	Experimental	Sedentary	Treadmill running
Krysciak et al., 2018	Standard	Wistar	Not specified	10–12 weeks	Young	Male and female	Not specified	Experimental	Not specified	Treadmill running
Kato et al., 2020	Standard	Wistar	56	11–24 days	Young	Male	Not specified	Experimental	Sedentary	Treadmill running
Botella et al., 2022	Standard (ZT0=08:00, ZT12=20:00)	Wistar	24	4 weeks	Young	Male	Not specified	Experimental	Not specified	Treadmill running
Pengam et al., 2023	Standard	Wistar	Not specified	4 weeks	Young	Male	Not specified	Experimental	Not specified	Treadmill running
Leise et al., 2013	Standard	C57BL/6	14	18–20 months	Old	Male	Not specified	Experimental	No wheel	Running wheel
Yasumoto et al., 2015	Standard	BALB/C	Not specified	5 weeks	Young	Not specified	Not specified	Experimental	No wheel	Running wheel
Bartling et al., 2017	Standard	C57BL/C	46	4-40 weeks	Mixed	Male and female	Not specified	Experimental	No wheel	Running wheel
Clark et al., 2015	Standard	Fischer344	70	Not specified	Not specified	Male	~148 g	Experimental	No wheel	Running wheel
Sheahan et al., 2015	Standard	C57BL/6	Not specified	7–10 weeks	Young	Male	Not specified	Experimental	Locked wheel	Running wheel
Tal-Krivisky et al., 2015	Standard	Sand rats	64	> 6 months	Young	Not specified	Not specified	Experimental	Not specified	Running wheel
Sah et al., 2017	Standard	C57BL/6	87	5–8 wee	Young	Male	Not specified	Experimental	No wheel	Running wheel
Thompson et al., 2016	Standard	Fischer344	16	50 days	Young	Male	200–230 g	Experimental	No wheel	Running wheel
Acevedo-Triana et al., 2017	Standard	Wistar	21	20–22 weeks	Old	Male	320–350 g	Experimental	No wheel	Running wheel
Toval et al., 2017	Standard	Sprague-Dawley	Not specified	20–22 weeks	Old	Male	320–350 g	Experimental	Untrained	Running wheel
Arnold et al., 2020	Standard	Fischer344	41	20–22 weeks	Old	Male	320–350 g	Two interventions experimental	Locked wheel	Running wheel
Stehle et al., 2021	Standard	Sprague-Dawley	28	7–8 weeks	Young	Male	180–220 g	Experimental	Locked wheel	Running wheel
Bilu et al., 2022	Short photoperiod	Sand rat	24	6–7 months	Young	Male	Not specified	Experimental	No wheel	Running wheel
Casanova-Vallve et al., 2022	Standard	C57BL/6J	24	9 weeks	Young	Male	Not specified	Experimental	No wheel	Running wheel
Sasaki et al., 2022	Standard	ICR	Not specified	8 weeks	Young	Male	Not specified	Experimental	Locked wheel	Running wheel
Dial et al., 2024	Standard	C57BL/6J	45	12 weeks	Young	Male	Not specified	Experimental	Locked wheel	Running wheel
Cao et al., 2023	Controlled light enclosures	Sprague- Dawley	55	12 months	Young	Not specified	Not specified	Experimental	No wheel	Running wheel
Ciorciari et al., 2025	Standard	C57BL/6J	20	90 days	Young	Male	Not specified	Experimental	Untrained	Swimming
Ramos-Filho et al., 2015	Standard	Wistar	20	4–5 months	Young	Female	220 g	Experimental	Untrained	Swimming
Gashi et al., 2020	Standard	Wistar	32	11–16 months	Old	Male	350–500 g	Experimental	Untrained	Swimming
Tunca et al., 2021	Standard	Sprague-Dawley	32	Not specified	Not specified	Male	350-500 g	Experimental	Untrained	Swimming
Amirazodi et al., 2022	Standard	Wistar	45	20 months	Old	Male	350–450 g	Experimental	Untrained	Swimming
Wen et al., 2022	Standard	Wistar	Not specified	Not specified	Not specified	Male	180–220 g	Experimental	Not specified	Swimming
Lin et al., 2024	Standard	C57BL/6J	6	3–18 months	Mixed	Male	Not specified	Experimental	Not specified	Swimming
Freitas et al., 2024	Standard	Wistar	20	8 weeks	Young	Male	323 g	Experimental	Active control (wheel running)	Swimming
Mehrabi et al., 2025	Standard	Wistar	60 initial / 45 included	20 months	Old	Male	400 g	Experimental	Untrained	HIIT swimming
Lovison et al., 2018	Standard	Wistar	24	Not specified	Not specified	Male	350–400 g	Experimental	Uninjured	Ladder climbing
Chi et al., 2020	Standard	Sprague-Dawley	104	8 weeks	Young	Male	Not specified	Experimental	Sedentary (acute/chronic)	Ladder climbing
Hubbard et al., 2022	Standard	Sprague-Dawley	11	Not specified	Not specified	Male	400 g	Experimental	Untrained	Downhill running
D'Hulst et al., 2022	Standard	C57BL/6J	Not specified	12–14 weeks	Young	Female	Not specified	Experimental	Sedentary	Resistance wheel
Hasegawa et al., 2018	Standard	Sprague-Dawley	40	10 weeks	Young	Male	Not specified	Experimental	Sedentary	Treadmill Running + Ladder climbing + Swimming

**Table 3:** Exercise protocols in included studies.

Study/Exercise type	Rodent species	Exercise protocol				
		Type	Frequency	Intensity	Time/Duration	Time of exercise
Aerobic Exercise						
Guo et al. (2012)	C57BL/6J	Treadmill Running	Three days per week	6 m/min for 30 mins at 5% incline	Short-term	Not considered
Ghanbari-Niaki and Rahmati-Ahmadabad (2013)	Wistar	Treadmill Running	Five days per week	25 m/min for 60 mins	Moderate-term	Not considered
Monaco et al. (2015)	Sprague-Dawley	Treadmill Running	Not mentioned	12 m/min for 60 mins at 5% incline	Not mentioned	Not considered
Lalanza et al. (2015)	Sprague-Dawley	Treadmill Running	Four to five days per week	Gradual increase up to 12 m/min	Moderate term	Not considered
Hortemo et al. (2016)	Sprague-Dawley	Treadmill Running	Six days per week	Initial at 15 m/min up to 25 m/min	Short-term	Not considered
Li et al. (2016)	Sprague-Dawley	Treadmill Running	Not mentioned	Initial at 10 m/min up to 15 m/min	Moderate-term	Not considered
Curzi et al. (2016)	Sprague-Dawley	Treadmill Running	Twice a day/ daily	Slow run - 15 m/min for 30 mins Fast run - 25 m/min fr 30 mins	Short-term	Not considered
Dalise et al. (2017)	Sprague-Dawley	Treadmill Running	Five days per week	80% of max capacity	Short-term	Not considered
Moshtagh et al. (2018)	Wistar	Treadmill Running	Not mentioned	Not mentioned	Short-term	Not considered
Aguiar et al. (2018)	C57BL/6J	Treadmill Running	Not mentioned	Not mentioned	Not mentioned	Not considered
Krysciak et al. (2018)	Wistar	Treadmill Running	Five days a week	Initial 10 m/min up to 40 m/min with intermittent acceleration	Moderate-term	Not considered
Kato et al. (2020)	Wistar	Treadmill Running	Five days per week	Initial 15 m/min for 30 min, up to 30 m/min for 90 min at a 5° incline.	Moderate-term	Considered
Botella et al. (2022)	Wistar	Treadmill Running	Not mentioned	80% of top speed of incremental test	Not mentioned	Not considered
Pengam et al. (2023)	Wistar	Treadmill Running	Five days per week	MICT^1^ – 50 mins running with 65% MAS HITT^2^ – Five times of 5 mins running at 85%-90% MAS^3^	Short-term	Not considered
Leise et al. (2013)	C57BL/6J	Running Wheel	Free access	Free access	Not mentioned	Not considered
Yasumoto et al. (2015)	BALB/C	Running Wheel	Free access	Free access	Short-term	Not considered
Bartling et al. (2017)	C57BL/C	Running Wheel	Free access	Free access	Short-term	Not considered
Clark et al. (2015)	Fischer344	Running Wheel	Free access	Free access	Short-term	Not considered
Sheahan et al. (2015)	C57BL/6J	Running Wheel	Five to six days per week	2-hour or 12-hour sessions	Not mentioned	Not considered
Tal-Krivisky et al. (2015)	Sand rats	Running Wheel	Free access	Free access	Not mentioned	Not considered
Sah et al. (2017)	C57BL/6J	Running Wheel	Free access	Free access	Not mentioned	Not considered
Thompson et al. (2016)	Fischer344	Running Wheel	Free access	Free access	Not mentioned	Not considered
Acevedo-Triana et al. (2017)	Wistar	Running Wheel	Five days per week	7 m/s for 30 mins	Not mentioned	Not considered
Toval et al. (2017)	Sprague-Dawley	Running Wheel	Not mentioned	Initial 9 m/min and gradual increase until failure	Short-term	Not considered
Arnold et al. (2020)	Fischer344	Running Wheel	Five days per week	Free access	Short-term	Not considered
Stehle et al. (2021)	Sprague-Dawley	Running Wheel	Not mentioned	Not mentioned	Short-term	Not considered
Bilu et al. (2022)	Sand rat	Running Wheel	Free access	Free access	Moderate-term	Not considered
Casanova-Vallve et al. (2022)	C57BL/6J	Running Wheel	Free access	Less than 20% of the maximum speed up to 50% maximum speed	Short to moderate-term	Not considered
Sasaki et al. (2022)	ICR	Running Wheel	Free access	Free access	Not mentioned	Not considered
Dial et al. (2024)	C57BL/6J	Running Wheel	Five days per week	6 hours of free access	Short-term	Considered
Cao and Gregoire (2023)	Sprague Dawley	Running Wheel	Five days per week	Initial 4 m/min for 1 hour/day up to 9 m/min for 2 hours/day	Long-term	Not considered
Ciorciari et al. (2025)	C57BL/6J	Running Wheel	Free access	Free access	Short-term (two weeks)	Not considered
Ramos-Filho et al. (2015)	Wistar	Swimming	Six days per week	Loaded 16% of body weight for 20 sec	Short-term	Not considered
Gashi et al. (2020)	Wistar	Swimming	Six days per week	50 mins gradually increased to 90 mins per day	Short-term	Not considered
Tunca et al. (2021)	Sprague-Dawley	Swimming	Five days per week	30 mins/ day	Short-term	Not considered
Amirazodi et al. (2022)	Wistar	Swimming	Three days per week	Loaded 9% of body weight for 14 to 20-sec	Short-term	Not considered
Wen et al. (2022)	Wistar	Swimming	Six days per week	Loaded 10% of body weight until exhaustion	Short-term	Not considered
Lin et al. (2024)	C57BL/6J	Swimming	Three days per week	First stage - 10 min/day Second stage – 15 min/day	Short-term	Not considered
Freitas et al. (2024)	Wistar	Swimming	Five days per week	10 min/day up to 2 hours/day	Long-term	Not considered
Mehrabi et al. (2025)	Wistar	Swimming	Three days per week	Loaded 9% of body weight gradualy increased by 1% weekly	Short-term	Not considered
Resistance Exercise						
Lovison et al. (2018)	Wistar	Ladder Climbing	Five days per week	100g of loaded weight	Not mentioned	Not considered
Chi et al. (2020)	Sprague-Dawley	Ladder Climbing	Three days per week	Acute: Set 1, 25% body weight, four reps Set 2, 50% body weight, four reps Set 3, 75% body weight, four reps Set 4, 100% body weight, six reps Chronic: Initial at 50% body weight, ten reps, 10% increment	Moderate-term	Considered
Hubbard et al. (2022)	Sprague-Dawley	Downhill Running	Three days per week	15° decline, 16 m/min, seven reps for 5 mins each	Short-term	Not considered
D'Hulst et al. (2022)	C57BL/6J	Resisted Running Wheel	Three days per week	60% resistance	Not mentioned	Not considered
Mixed Exercise						
Lee et al. (2017)	Wistar	Treadmill Running + Jumping	Five days per week	Treadmill Running: Initial at 12 m/min for 30 mins up to 22 m/min for 60 mins Jumping: 100 jumps/10 min and 25 cm height, up to 40 cm	Moderate-term	Not considered
Hasegawa et al. (2018)	Sprague-Dawley	Treadmill Running + Swimming + Ladder Climbing	Treadmill Running - Five days per week Swimming - Four days per week Ladder Climbing - Three days per week	Treadmill Running - Initial 15 m/min up to 30 m/min Swimming- Not Mentioned Ladder Climbing- 80° incline, four reps/set for three sets	Treadmill Running – Moderate-term Swimming – Short-term Ladder Climbing – Moderate-term	Not considered

^1^HIIT – High-intensity interval training.^2^MICT – Moderate-intensity continuous training.^3^MAS – Maximal aerobic speed.

### 3.1 Outcomes

#### 3.1.1 Rodent species

The most frequently employed rodent species in the evaluated studies were Wistar and Sprague Dawley rats (n=15 and n=13, respectively), followed by C57BL/6J mice (n=11). Three studies (n=3) used Fischer-Brown 344 rats, while two studies used sand rats (n=2). ICR and BALB/C mice were used in only one study (n=1).

#### 3.1.2 Exercise protocol

##### 3.1.2.1 Exercise frequency

Among the 46 studies investigating exercise interventions, including aerobic and resistance modalities, 23 implemented structured exercise protocols with a frequency of three to six days per week (Guo et al., 2012; Ghanbari-Niaki and Rahmati-Ahmadabad, 2013; Lalanza et al., 2015; Ramos-Filho et al., 2015; Hortemo et al., 2016; Acevedo-Triana et al., 2017; Lee et al., 2017; Hasegawa et al., 2018; Krysciak et al., 2018; Arnold et al., 2020; Chi et al., 2020; Gashi et al., 2020; Kato et al., 2020; Tunca et al., 2021; Amirazodi et al., 2022; Hubbard et al., 2022; Wen et al., 2022; Cao and Gregoire, 2023; Pengam et al., 2023; Dial et al., 2024; Freitas et al., 2024; Mehrabi et al., 2025). Another eleven studies allowed animals free access to exercise equipment, mainly running wheels (Leise et al., 2013; Clark et al., 2015; Tal-Krivisky et al., 2015; Yasumoto et al., 2015; Thompson et al., 2016; Bartling et al., 2017; Sah et al., 2017; Arnold et al., 2020; Bilu et al., 2022; Casanova-Vallve et al., 2022; Sasaki et al., 2022; Cao and Gregoire, 2023; Dial et al., 2024; Ciorciari et al., 2025). Eight studies did not report exercise frequency (Monaco et al., 2015; Li et al., 2016; Toval et al., 2017; Aguiar et al., 2018; Moshtagh et al., 2018; Stehle et al., 2021; Botella et al., 2022; D'Hulst et al., 2022).

##### 3.1.2.2 Exercise intensity

Regarding intensity, nine studies employed fixed parameters, such as treadmill speeds ranging from 6 to 40 m/min or ladder climbing with fixed loads of 100 g (Guo et al., 2012; Monaco et al., 2015; Curzi et al., 2016; Acevedo-Triana et al., 2017; Lovison et al., 2018). Three studies prescribed intensity based on maximal aerobic speed (MAS), ranging from 65% to 95%, with durations ranging from 5 to 50 minutes (Dalise et al., 2017; Botella et al., 2022; Pengam et al., 2023). Two studies restricted voluntary access to 2–12 hours per day (Sheahan et al., 2015; Dial et al., 2024).

Several studies employed progressive or incremental intensity protocols. For treadmill running or wheel access, intensities started as low as 4 m/min and increased up to 30 m/min, with durations of 30 minutes to 2 hours (Lalanza et al., 2015; Hortemo et al., 2016; Li et al., 2016; Lee et al., 2017; Hasegawa et al., 2018; Krysciak et al., 2018; Kato et al., 2020; Cao and Gregoire, 2023) or continued until exhaustion (Toval et al., 2017). Ladder climbing intensity was progressively increased from 25% to 100% of body weight (Hasegawa et al., 2018; Chi et al., 2020), while one study increased speed from 20% to 50% of maximum speed (Casanova-Vallve et al., 2022). Four studies did not report exercise intensity (Aguiar et al., 2018; Moshtagh et al., 2018; Gashi et al., 2020; Stehle et al., 2021).

Swimming interventions included both loaded and unloaded sessions. Loaded swimming ranged from 9% to 16% of body weight, lasting 14–20 seconds (Ramos-Filho et al., 2015; Amirazodi et al., 2022), until exhaustion (Wen et al., 2022), or gradually increasing by 1% of body weight per week (Mehrabi et al., 2025). Unloaded swimming sessions lasted from 10 minutes to 3 hours per day (Tunca et al., 2021; Freitas et al., 2024; Lin et al., 2024), and two studies did not specify swimming intensity (Hasegawa et al., 2018; Gashi et al., 2020). Jumping exercise was reported in one study, with 100 jumps over 10 minutes at heights increasing from 25 to 40 cm (Lee et al., 2017).

##### 4.1.2.3 Exercise duration

Regarding intervention duration, 17 studies were short-term (<8 weeks) (Guo et al., 2012; Clark et al., 2015; Ramos-Filho et al., 2015; Yasumoto et al., 2015; Curzi et al., 2016; Hortemo et al., 2016; Bartling et al., 2017; Dalise et al., 2017; Moshtagh et al., 2018; Gashi et al., 2020; Stehle et al., 2021; Tunca et al., 2021; Amirazodi et al., 2022; Wen et al., 2022; Cao and Gregoire, 2023; Pengam et al., 2023; Ciorciari et al., 2025; Mehrabi et al., 2025), 13 studies were moderate-term (≥8 weeks) (Ghanbari-Niaki and Rahmati-Ahmadabad, 2013; Lalanza et al., 2015; Li et al., 2016; Lee et al., 2017; Hasegawa et al., 2018; Krysciak et al., 2018; Chi et al., 2020; Kato et al., 2020; Bilu et al., 2022; Casanova-Vallve et al., 2022; Cao and Gregoire, 2023; Freitas et al., 2024; Lin et al., 2024), and two studies implemented long-term (≥12 weeks) interventions (Cao and Gregoire, 2023; Freitas et al., 2024). Ten studies did not report intervention duration (Leise et al., 2013; Monaco et al., 2015; Sheahan et al., 2015; Tal-Krivisky et al., 2015; Thompson et al., 2016; Acevedo-Triana et al., 2017; Sah et al., 2017; Toval et al., 2017; Aguiar et al., 2018; Botella et al., 2022).

#### 3.1.3 Changes in structural and body composition

Twenty-nine studies (n=29) reported significant changes in body weight, composition, tissues and organs following aerobic exercise. Body weight reductions ranged from 10% to 41%, representing substantial decreases relative to the control and comparable groups, respectively (Aguiar et al., 2018; Moshtagh et al., 2018; Arnold et al., 2020; Sasaki et al., 2022), whereas fat mass decreased by 25% to nearly 48%, a marked reduction relative to the control and comparable groups, respectively (Guo et al., 2012; Ramos-Filho et al., 2015; Yasumoto et al., 2015; Hasegawa et al., 2018; Kato et al., 2020; Wen et al., 2022). Muscle mass increased by 8% to 33%, indicating significant hypertrophy compared to the control (Hasegawa et al., 2018) and improvements in motor unit functionality were observed up to 27%, reflecting considerable neuromuscular adaptation compared to the control (Krysciak et al., 2018).

Adipose tissue reductions ranged from 15% to 16% following exercise interventions, demonstrating consistent but modest improvements compared to the control (Yasumoto et al., 2015). Five studies reported significant improvements in liver weight, bone morphology, brain cells and heart weight (Li et al., 2016; Sah et al., 2017; Moshtagh et al., 2018; Bilu et al., 2022; Dial et al., 2024). However, three studies reported no significant changes in brain tissue (Tunca et al., 2021) or in bone and heart morphology (Tunca et al., 2021; Cao and Gregoire, 2023; Freitas et al., 2024). One study reported improvements in lens tissue (Lin et al., 2024).

In resistance exercise, one study reported a significant muscle mass increase of up to 19%, along with a nearly 26% reduction in fat mass, reflecting substantial compositional changes compared to the control group (Chi et al., 2020). Concerning tissue changes, two studies focused on muscle tissue, with one reporting no changes (Hubbard et al., 2022) and the other reporting structural improvements (Lovison et al., 2018).

The structural and compositional adaptations to exercise appear to be age-dependent. The majority of evidence in young rodents consistently demonstrates robust reductions in body weight (between 10-41%) and fat mass (between 25-48%), alongside increases in muscle mass (between 8-33%), suggesting high metabolic plasticity and pronounced anabolic responsiveness (Ramos-Filho et al., 2015; Yasumoto et al., 2015; Aguiar et al., 2018; Hasegawa et al., 2018; Krysciak et al., 2018; Kato et al., 2020; Sasaki et al., 2022). These findings, largely derived from young cohorts, indicate a high degree of metabolic plasticity and anabolic responsiveness in early life. Additional organ-level adaptations, including changes in liver, bone, brain, and cardiac tissues, were also predominantly reported in young or unspecified-age models (Sah et al., 2017; Moshtagh et al., 2018; Bilu et al., 2022; Dial et al., 2024). In contrast, only a limited subset of studies explicitly investigated aged rodents, constraining definitive conclusions regarding structural adaptations in later life. Available evidence suggests that older animals retain partial responsiveness to aerobic exercise, particularly in terms of fat mass reduction (Guo et al., 2012) and organ-level adaptations, including improvements in hepatic and cardiac parameters (Li et al., 2016; Arnold et al., 2020). Studies including both young and old animals or unspecified age cohorts suggest that aging may blunt certain adaptations like muscle hypertrophy, while cardiometabolic benefits remain relatively preserved (Bartling et al., 2017). Overall, the evidence indicates that early-life exercise yields larger body composition shifts, whereas in older rodents, exercise may exert more protective or maintenance-oriented effects rather than dramatic structural remodelling.

Forced exercise protocols demonstrated more uniform and reproducible improvements in body composition and tissue morphology across studies. The standardised control of intensity, duration, and workload likely contributes to this consistency, ensuring that animals receive a sufficient stimulus to induce measurable structural adaptation (Guo et al., 2012; Ramos-Filho et al., 2015; Li et al., 2016; Aguiar et al., 2018; Hasegawa et al., 2018; Krysciak et al., 2018; Chi et al., 2020; Kato et al., 2020; Freitas et al., 2024; Lin et al., 2024). In contrast, voluntary exercise paradigms produced more heterogeneous findings, with improvements interspersed with null effects (Sheahan et al., 2015; Yasumoto et al., 2015; Thompson et al., 2016; Acevedo-Triana et al., 2017; Bartling et al., 2017; Bilu et al., 2022; Sasaki et al., 2022; Cao and Gregoire, 2023; Dial et al., 2024). This variability may reflect differences in self-selected activity levels and inter-individual engagement, leading to less predictable dose exposure. While voluntary exercise remains beneficial, the magnitude and reliability of compositional and organ-level adaptations appear less consistent compared to forced protocols. Collectively, this evidence suggests that modality primarily influences the reproducibility and consistency of structural changes, with forced paradigms generating more robust and standardised adaptations.

There have been limited studies on exercise timing. Kato et al. reported in an aerobic exercise that late active exercise resulted in greater weight loss than early active exercise (Kato et al., 2020). Another study on cardiac health suggested that early active exercise was associated with significant cardiac hypertrophy (Dial et al., 2024). However, a study on resistance exercise reported no significant difference in muscle mass between early and late active exercise phases (Chi et al., 2020). **Table 4** summarises the changes in the structural and body composition.

**Table 4:** Changes in the structural and body composition.

Exercise type	Domain	Key findings	Study ref.
Aerobic Exercise	Body Weight	Reduction of 10–41%	(Aguiar et al., 2018; Moshtagh et al., 2018; Arnold et al., 2020; Sasaki et al., 2022; Freitas et al., 2024)
	Fat Mass	Reduction of 25–48%	(Guo et al., 2012; Ramos-Filho et al., 2015; Yasumoto et al., 2015; Hasegawa et al., 2018; Kato et al., 2020; Wen et al., 2022)
	Muscle Mass	Improved by 8–33%	(Hasegawa et al., 2018)
	Motor Unit Functionality	Improved by ~27%	(Krysciak et al., 2018)
	Adipose Tissue	Reduction of 15–16%	(D'Hulst et al., 2022)
	Organ & Tissue Changes	Improved liver weight, bone morphology, brain cells and heart weight	(Li et al., 2016; Sah et al., 2017; Moshtagh et al., 2018; Bilu et al., 2022; Dial et al., 2024)
		No changes in brain tissue, bone, or heart morphology	(Tunca et al., 2021; Cao and Gregoire, 2023; Freitas et al., 2024)
		Improved lens tissue	(Lin et al., 2024)
Resistance Exercise	Body Composition	Improved muscle mass (up to 19%); Reduction in fat mass (~26%)	(Chi et al., 2020)
	Muscle Tissue	No changes (1 study); Improved structural adaptations (1 study)	(Lovison et al., 2018; Hubbard et al., 2022)
Exercise Timing	Weight Loss (Aerobic)	Greater reduction in late vs. early phase	(Kato et al., 2020)
	Cardiac Health (Aerobic)	Improved cardiac hypertrophy in the early active phase	(Dial et al., 2024)
	Muscle Mass (Resistance)	No changes between phases	(Chi et al., 2020)

#### 3.1.4 Changes in neurobehavior

Only aerobic exercise studies (n=7) reported changes in neurobehavior following interventions. The behavioural tests used included the shuttle box, novel object recognition test, Morris water maze and t-maze for assessing cognitive function. Anxiety levels were assessed via the elevated plus maze and forced swim test. Adaptation and responsiveness were assessed via the cold-plantar assay, von Frey, Hargreaves and hotplate tests. The social interaction test was used to assess social interaction ability, whereas biotelemetry was used to monitor sleep patterns.

Four studies assessed cognitive function, with three showing significant improvements (Lalanza et al., 2015; Tunca et al., 2021; Bilu et al., 2022), while one found no changes (Acevedo-Triana et al., 2017). Two studies reported significant improvements in anxiety and depression symptoms, leading to better emotional resilience (Tal-Krivisky et al., 2015; Bilu et al., 2022). Additionally, one study found a significant improvement in sleep quality (Thompson et al., 2016), while another observed no changes in adaptability and response (Sheahan et al., 2015). Improvements in social interaction were also noted in one study (Tal-Krivisky et al., 2015).

It is important to note that although resistance exercise was included as a modality of interest in this scoping review, none of the eligible rodent studies specifically investigated the effects of resistance training on neurobehavioral outcomes. This absence does not necessarily indicate a lack of effect but rather reflects a gap in the preclinical literature. The resistance exercise research in rodents has primarily focused on musculoskeletal, metabolic and hormonal adaptations, with relatively limited exploration of cognitive, emotional or behavioural endpoints.

Comparatively, different age groups demonstrated a differential pattern of neurobehavioral responsiveness. In young rodents, aerobic exercise was generally associated with improvements in hippocampal-dependent cognitive tasks such as Morris water maze, novel object recognition and affective measures, indicating a high degree of neuroplastic responsiveness during early life (Lalanza et al., 2015; Bilu et al., 2022). These improvements were often accompanied by enhancements in affective domains, including reduced anxiety- and depression-like behaviours (Tal-Krivisky et al., 2015; Bilu et al., 2022), and improved sleep quality in some cases (Thompson et al., 2016), suggesting broad neurobehavioral benefits extending beyond cognition. In contrast, the limited number of studies including aged rodents (Acevedo-Triana et al., 2017) reported less consistent cognitive improvements, with some null findings in learning and memory tasks, indicating that ageing may attenuate exercise-induced cognitive enhancement. Despite this attenuation, older animals appear to retain responsiveness in affective and resilience-related domains, with improvements in anxiety- and depression-like behaviours persisting even when cognitive gains are modest or variable (Acevedo-Triana et al., 2017). This pattern suggests an age-dependent shift in neurobehavioral adaptation, whereby exercise in youth is associated with enhancement and expansion of cognitive and emotional function, while in older animals it may primarily support maintenance of function and emotional stability, rather than driving substantial performance gains.

Similarly, forced and voluntary aerobic paradigms demonstrate distinct but overlapping neurobehavioral profiles. Forced aerobic exercise showed more consistent improvements in cognitive outcomes, particularly in structured learning and memory tasks (Lalanza et al., 2015; Tunca et al., 2021). The standardised intensity and duration inherent to forced paradigms may provide a sufficient and reproducible physiological stimulus to induce robust neuroplastic changes. However, the potential contribution of stress exposure cannot be excluded, as stress-related arousal may interact with learning and affective outcomes. In contrast, voluntary exercise yielded more heterogeneous findings, with benefits observed in emotional regulation, social interaction, and cognition in some cases but with greater variability across studies (Sheahan et al., 2015; Tal-Krivisky et al., 2015; Thompson et al., 2016; Acevedo-Triana et al., 2017; Bilu et al., 2022). This variability likely reflects differences in self-selected running volume and inter-individual activity levels. Overall, these findings suggest that modality influences the consistency and domain specificity of neurobehavioral adaptations that forced exercise appears to produce more reliable cognitive improvements, whereas voluntary exercise may preferentially support affective and social domains. The absence of resistance training studies examining neurobehavioral endpoints highlights a modality-specific gap in the preclinical literature.

None of the included studies explored the impact of specific exercise timing on neurobehavioral changes. The neurobehavioral effects of exercise interventions are summarised in **Table 5**.

**Table 5:** Changes in the neurobehavior.

Exercise type	Domain	Assessment tools	Key findings	Study ref.
Aerobic Exercise	Cognitive Function	Shuttle box, novel object recognition, Morris water maze, t-maze	Improved cognition; No changes	(Lalanza et al., 2015; Acevedo-Triana et al., 2017; Tunca et al., 2021; Bilu et al., 2022)
	Anxiety & Depression	Elevated plus maze, forced swim test	Improved symptoms (better emotional resilience)	(Tal-Krivisky et al., 2015; Bilu et al., 2022)
	Adaptability & Responsiveness	Cold-plantar, von Frey, Hargreaves, hotplate	No changes	(Sheahan et al., 2015)
	Social Interaction	Social interaction test	Improved	(Tal-Krivisky et al., 2015)
	Sleep Quality	Biotelemetry monitoring	Improved	(Thompson et al., 2016)
Exercise Timing	–	–	No studies reported	–

#### 3.1.5 Changes in physiological function

Twenty-five studies (n=25) reported physiological outcomes following aerobic exercise, focusing on aspects such as physical performance, respiratory and metabolic function, muscle strength, cardiovascular function, recovery and neurotransmission. Fourteen studies reported significant improvements in physical performance, with increases of up to sixfold relative to the control and comparable group, respectively (Leise et al., 2013; Ramos-Filho et al., 2015; Tal-Krivisky et al., 2015; Thompson et al., 2016; Dalise et al., 2017; Toval et al., 2017; Aguiar et al., 2018; Hasegawa et al., 2018; Tunca et al., 2021; Amirazodi et al., 2022; Casanova-Vallve et al., 2022; Wen et al., 2022; Pengam et al., 2023). One study reported no significant changes in performance (Guo et al., 2012; Hüttemann et al., 2012). One study reported significant improvements in muscle function and strength (Krysciak et al., 2018) while one did not demonstrate any changes (Curzi et al., 2016). Two other studies demonstrated significant improvements in respiratory function and metabolism, with increases in O_2_ consumption of up to 17.1% relative to each of the comparable groups (Guo et al., 2012; Kato et al., 2020).

In terms of cardiovascular function, one study reported a significant improvement in arterial stiffness (Hasegawa et al., 2018), whereas other studies reported no significant changes in heart rate (Thompson et al., 2016; Hasegawa et al., 2018) or blood pressure (Hasegawa et al., 2018). Three studies reported significant enhancement of thermoregulation, which may lead to better recovery (Yasumoto et al., 2015; Thompson et al., 2016; Aguiar et al., 2018). Additionally, two studies reported no changes in neurotransmission and neurophysiology (Sheahan et al., 2015; Sah et al., 2017). One previous study has demonstrated that aerobic exercise significantly facilitates a faster circadian rhythm recovery by promoting significantly quicker realignment compared to non-exercise conditions following disruption (Ciorciari et al., 2025).

In resistance exercise, one study reported a significant increase in muscle strength (Chi et al., 2020), whereas another reported no significant changes (Hubbard et al., 2022). Regarding exercise timing, one study reported that body metabolism was significantly greater during late active exercise than during the early active phase (Kato et al., 2020). One study reported no differences in muscle strength between the two exercise phases (Chi et al., 2020). **Table 6** presents the physiological changes observed following exercise interventions.

**Table 6:** Changes in the physiological function.

Exercise type	Domain	Key findings	Study ref.
Aerobic Exercise	Physical Performance	Improved in 14 studies; No changes in 2 studies	(Guo et al., 2012; Leise et al., 2013; Ramos-Filho et al., 2015; Tal-Krivisky et al., 2015; Thompson et al., 2016; Dalise et al., 2017; Toval et al., 2017; Aguiar et al., 2018; Hasegawa et al., 2018; Tunca et al., 2021; Amirazodi et al., 2022; Casanova-Vallve et al., 2022; Wen et al., 2022; Pengam et al., 2023)
	Muscle Strength	Improved muscle function and strength (1 study), No changes (1 study)	(Curzi et al., 2016; Krysciak et al., 2018)
	Respiratory & Metabolic Function	Improved O_2_ consumption (up to 17.1%)	(Guo et al., 2012; Kato et al., 2020)
	Cardiovascular Function	Improved arterial stiffness (1 study); No changes in HR and BP (2 studies)	(Hortemo et al., 2016; Thompson et al., 2016)
	Recovery & Thermoregulation	Improved thermoregulation (3 studies); Improved circadian rhythm recovery	(Yasumoto et al., 2015; Thompson et al., 2016; Aguiar et al., 2018; Dial et al., 2024; Ciorciari et al., 2025)
	Neurotransmission & Neurophysiology	No changes	(Sheahan et al., 2015; Sah et al., 2017)
Resistance Exercise	Muscle Strength	Improved (1 study); No changes (1 study)	(Chi et al., 2020; Hubbard et al., 2022)
Exercise Timing	Metabolism	Improved in the late vs. early active phase	(Kato et al., 2020)
	Muscle Strength	No changes between phases	(Chi et al., 2020)

The physiological benefits of exercise appear predominantly driven by studies conducted in young animals. The large majority of included studies were performed exclusively in young rodents. These consistently demonstrated improvements in physical performance up to sixfold increases (Tal-Krivisky et al., 2015; Thompson et al., 2016; Dalise et al., 2017; Aguiar et al., 2018; Amirazodi et al., 2022; Casanova-Vallve et al., 2022; Pengam et al., 2023), metabolic efficiency such as an increase in CO_2_ consumption up to 17.1% (Kato et al., 2020), thermoregulation (Yasumoto et al., 2015; Aguiar et al., 2018), and muscle function (Krysciak et al., 2018; Chi et al., 2020) as well as recovery (Hasegawa et al., 2018; Ciorciari et al., 2025). In contrast, only a small number of studies examined older animals, limiting firm conclusions in later life. However, available evidence suggests that older rodents retain responsiveness to exercise stimuli, particularly in metabolic and cardiovascular domains, including improvements in oxygen consumption (Guo et al., 2012) and preserved gains in physical performance in some cases (Toval et al., 2017). However, these effects appear less consistent and generally attenuated compared to young cohorts, with greater variability and fewer robust improvements across studies. This imbalance in age representation indicates that current evidence is heavily weighted toward early-life physiological plasticity, while the translational relevance to ageing populations remains underexplored. The limited data in aged models also prevent clear conclusions regarding whether exercise mitigates age-related physiological decline or merely preserves baseline function.

Forced exercise protocols were consistently associated with measurable improvements in physical performance and metabolic outcomes, including increased oxygen consumption and thermoregulation (Ramos-Filho et al., 2015; Dalise et al., 2017; Aguiar et al., 2018; Krysciak et al., 2018; Chi et al., 2020; Kato et al., 2020; Tunca et al., 2021; Amirazodi et al., 2022; Wen et al., 2022; Pengam et al., 2023). These structured protocols may produce more standardised physiological overload, thereby yielding clearer adaptations. In contrast, voluntary exercise studies demonstrated comparable directional trends in physiological enhancement but with greater variability, potentially reflecting differences in self-selected intensity and engagement (Leise et al., 2013; Tal-Krivisky et al., 2015; Yasumoto et al., 2015; Thompson et al., 2016; Toval et al., 2017; Casanova-Vallve et al., 2022; Dial et al., 2024; Ciorciari et al., 2025). Particularly, null findings in cardiovascular parameters (heart rate and blood pressure) were observed primarily within forced protocols, suggesting that certain physiological systems may be less sensitive to exercise dose or require longer intervention durations (Hasegawa et al., 2018). These findings suggest that forced exercise produces more consistent and reproducible adaptations, whereas voluntary exercise better reflects ecologically relevant but more variable responses, particularly in ageing contexts.

The evidence on chrono-exercise suggests that timing may modulate physiological outcomes in an age- and domain-specific manner, although data remain limited and largely derived from young cohorts. For example, greater metabolic responses during late active-phase exercise were observed in a young rodent study (Kato et al., 2020), while no timing-dependent differences in muscle strength were found in resistance training (Chi et al., 2020).

#### 3.1.6 Changes in biochemical regulation

Significant changes in biochemical regulation have been reported to influence a variety of physiological functions, including physical and muscle performance, cardiovascular and metabolic health, energy production and neurobehavioral outcomes such as cognitive and mental health. Several studies have documented significant enhancements in physical and muscle performance following aerobic exercise. These improvements are associated with increased expression of key genes, including PCG-1α, nuclear factor erythroid 2-related factor 1 and 2 (Nrf1, Nrf2), and Mfn2 mRNA (Dalise et al., 2017; Pengam et al., 2023). These increases are critical for muscle energy metabolism. Additionally, a significant elevation in the mitochondrial DNA-to-nuclear DNA ratio (mtDNA/DNA) (Guo et al., 2012), increased production of mitofusin 1 (Mfn1), mitofusin 2 (Mfn2) proteins, and dynamin-like protein (Krysciak et al., 2018; Pengam et al., 2023), O-GlcNAcylation proteins (Hortemo et al., 2016) and a marked increase in complex V protein expression (Dial et al., 2024) contribute to significant mitochondrial biogenesis.

Moreover, elevated levels of enzymes such as adipose triglyceride lipase (ATGL), HSL, perilipin1 and PPAR2 (Kato et al., 2020), as well as significant increases in hormones such as leptin and ghrelin (Yasumoto et al., 2015), have also been reported, demonstrating their involvement in energy metabolism. Substantial improvements in cardiovascular and metabolic health were reported in four studies. These studies revealed significant adenosine triphosphate (ATP)-binding cassette subfamily C member 2 (ABCC2) mRNA overexpression, which is important for cardiac health (Ghanbari-Niaki and Rahmati-Ahmadabad, 2013). Additionally, increased synthesis of the fatty acid translocase/cluster of differentiation 36 (FAT/CD36) enzyme, which is strongly associated with lipid metabolism, has been noted (Monaco et al., 2015). Enhanced citrate synthase activity, contributing to greater metabolic efficiency, has also been reported (Hasegawa et al., 2018). Significant increases in high-density lipoprotein cholesterol (HDL-C) and reactive oxygen metabolite-derived compounds (d-ROMs) help maintain oxidative balance (Ghanbari-Niaki and Rahmati-Ahmadabad, 2013), along with elevated levels of microtubule-associated proteins (1A/1B light chain 3BLC3B), which are crucial for autophagy (Botella et al., 2022). One previous study demonstrated a marked improvement in antioxidant defence by enhancing SIRT4 by 1.63-fold as well as SOD1 and SOD2 by 1.74- and 1.43-fold, respectively (Mehrabi et al., 2025).

Six studies reported significant improvements in cognitive and mental health. These studies revealed elevated levels of hormones that enhance cognitive function, including brain-derived neurotrophic factor (BDNF) (Tunca et al., 2021), adrenocorticotropic hormone (ACTH) (Lalanza et al., 2015), dopamine (Clark et al., 2015) and serotonin (Arnold et al., 2020). Additionally, cognitive benefits were attributed to significantly reduced levels of Nogo-A protein (Stehle et al., 2021), increased CREB protein expression (Tunca et al., 2021) and an improved NAD+/NADH ratio (Amirazodi et al., 2022) after exercise intervention.

Resistance exercise also induces significant biochemical regulatory changes that enhance physical and muscle performance. Increased expression of genes such as activating transcription factor 4 (ATF4) and greater mechanistic target of rapamycin complex 1 (mTORC1) sensitivity have been reported to improve muscle function (D'Hulst et al., 2022). Elevated levels of testosterone and interleukin-6 (IL-6) following resistance exercise are significantly associated with better physical performance (Chi et al., 2020). However, one study reported no measurable improvement following the exercise intervention (Hubbard et al., 2022). **Table 7** shows changes in biochemical regulation.

**Table 7:** Changes in the biochemical regulation.

Exercise type	Domain	Key molecular/Protein/Enzyme changes	Reported outcomes	Study Ref.
Aerobic Exercise	Physical & Muscle Performance	Improved PGC-1α, Nrf1, Nrf2, Mfn2 mRNA; Improved mtDNA/nDNA ratio; Improved Mfn1, Mfn2, Dynamin-like protein; Improved O-GlcNAcylation proteins; Improved Complex V protein	Improved mitochondrial biogenesis, energy metabolism, and muscle performance	(Guo et al., 2012; Curzi et al., 2016; Hortemo et al., 2016; Dalise et al., 2017; Krysciak et al., 2018; Pengam et al., 2023; Dial et al., 2024)
	Energy Metabolism	Improved ATGL, HSL, Perilipin1, PPAR2; Improved leptin, ghrelin	Improved lipid mobilization and energy regulation	(Yasumoto et al., 2015; Kato et al., 2020)
	Cardiovascular & Metabolic Health	Improved ABCC2 mRNA; Improved FAT/CD36; Improved citrate synthase activity; Improved HDL-C and d-ROMs; Improved LC3B protein	Improved cardiac function, lipid metabolism, oxidative balance, and autophagy	(Ghanbari-Niaki and Rahmati-Ahmadabad, 2013; Monaco et al., 2015; Hasegawa et al., 2018; Botella et al., 2022)
	Antioxidant Defense	Improved SIRT4, SOD1, SOD2	Improved antioxidant defense (SIRT4: 1.63-fold; SOD1: 1.74-fold; SOD2: 1.43-fold)	(Mehrabi et al., 2025)
	Cognitive & Mental Health	Improved BDNF, ACTH, dopamine, serotonin; Reduced Nogo-A; Improved CREB; Improved NAD^+^/NADH ratio	Improved cognition, mood, and neuroprotection	(Clark et al., 2015; Lalanza et al., 2015; Arnold et al., 2020; Stehle et al., 2021; Tunca et al., 2021; Amirazodi et al., 2022)
Resistance Exercise	Physical & Muscle Performance	Improved ATF4 expression; Improved mTORC1 sensitivity; Improved testosterone and IL-6; No changes	Improved muscle adaptation and strength	(Chi et al., 2020; D'Hulst et al., 2022; Hubbard et al., 2022; Wen et al., 2022)
Exercise Timing	Energy Metabolism	Improved HSL, Perilipin1, AKAP150, PKA RIIβ in late phase	Improved protein synthesis for energy production in late phase	(Kato et al., 2020)
	Inflammation	Improved IL-6 in the late active phase	More pronounced inflammatory response	(Chi et al., 2020)
	Mitochondrial Function	Improved Complex V protein in the late phase; No changes in IGF1 mRNA	Improved mitochondrial biogenesis; No effect on hypertrophy	(Dial et al., 2024)

In young rodents, exercise consistently upregulated genes and proteins related to mitochondrial biogenesis and energy metabolism (PGC-1α, Nrf1/2, Mfn1/2) (Krysciak et al., 2018), oxidative phosphorylation (Complex V) (Dial et al., 2024), autophagy (LC3B) (Botella et al., 2022), and antioxidant defence (SOD1/2, SIRT4) (Pengam et al., 2023), alongside neuroplasticity markers such as BDNF (Dalise et al., 2017) and CREB (Casanova-Vallve et al., 2022). These coordinated adaptations suggest a broad enhancement of metabolic flexibility and neuroenergetic efficiency in early life. In contrast, fewer studies of older rodents indicate that aerobic exercise still enhances mitochondrial proteins, lipid metabolism enzymes (e.g., FAT/CD36) (Monaco et al., 2015), and antioxidant systems (Guo et al., 2012; Mehrabi et al., 2025) as well as hormone secretion for neuron improvement (Arnold et al., 2020). However, the magnitude and diversity of molecular changes appear more selective and often oriented toward restoring redox balance and metabolic homeostasis rather than inducing robust anabolic or neurotrophic upregulation. Collectively, these findings suggest that exercise in young rodents drives expansive metabolic remodelling and neuroplastic signalling, whereas in older rodents it primarily acts as a compensatory mechanism to counteract age-related mitochondrial decline and oxidative stress.

Forced exercise, which predominates in the dataset, is associated with more consistent and robust biochemical responses. Multiple studies reported significant enhancements in metabolic regulation, mitochondrial activity, and oxidative stress balance (Lee et al., 2017; Aguiar et al., 2018; Hasegawa et al., 2018; Krysciak et al., 2018; Kato et al., 2020; Pengam et al., 2023; Freitas et al., 2024). These adaptations often include improved antioxidant defence, increased enzymatic activity, and enhanced energy metabolism. However, several studies (Lalanza et al., 2015; Gashi et al., 2020) also indicate that forced protocols may induce stress-related responses, which could influence neuroendocrine and inflammatory pathways. In contrast, voluntary exercise demonstrates beneficial but more variable adaptations. A few studies report improvements in metabolic and neurobiological markers, including enhanced antioxidant capacity and favourable biochemical regulation (Ramos-Filho et al., 2015; Yasumoto et al., 2015; Dalise et al., 2017; Bilu et al., 2022). Additionally, voluntary paradigms are frequently associated with reduced stress responses and improved behavioural and neuroplasticity-related outcomes (Stehle et al., 2021; Casanova-Vallve et al., 2022). However, the magnitude of these effects is less consistent, likely due to variability in individual activity levels and exercise engagement. The findings suggest that forced exercise provides more reproducible and measurable biochemical adaptations, whereas voluntary exercise offers greater physiological relevance and reduced stress interference, particularly in studies involving behavioural or ageing-related outcomes. The imbalance in study representation within the dataset, where forced exercise is more frequently employed, indicates that current biochemical evidence is largely driven by structured exercise models, potentially underrepresenting the effects of spontaneous physical activity.

The effects of exercise timing have also been explored in several studies. One study reported that protein synthesis related to energy production, including hormone-sensitive lipase (HSL), perilipin1, A-kinase anchoring protein 150 (AKAP150) and regulatory subunit IIβ of protein kinase A (RIIβ), was significantly greater during the late active exercise phase than during the early active phase (Kato et al., 2020). Another study reported significantly elevated levels of the inflammatory hormone IL-6 during late active exercise, suggesting a stronger inflammatory response (Chi et al., 2020). Additionally, Dial et al. (2024) reported that late active exercise significantly increased mitochondrial protein expression, particularly in Complex V, compared with the early active phase. However, no significant difference was observed in IGF1 mRNA levels associated with muscle hypertrophy (Dial et al., 2024).

## 4 Discussion

***Bridging the Outcomes: The Impact of Exercise on Brain-Muscle Cross Talk and Its Structural and Body Composition Improvement, Neurobehaviour Adaptation, Physiological Changes and Biochemical Regulation.***

Aerobic exercise is particularly effective for improving cardiovascular health and metabolic efficiency. It reduces fat mass, increases lean muscle mass, and lowers overall body weight, thereby helping to prevent obesity and related metabolic disorders (Guo et al., 2012; Ramos-Filho et al., 2015; Yasumoto et al., 2015; Aguiar et al., 2018; Hasegawa et al., 2018; Moshtagh et al., 2018; Nystoriak and Bhatnagar, 2018; Arnold et al., 2020; Chi et al., 2020; Kato et al., 2020; Sasaki et al., 2022; Wen et al., 2022). These systemic effects are supported by cellular adaptations, including increased mitochondrial density and oxidative enzyme activity, which enhance energy metabolism and cardiovascular performance (Guo et al., 2012; Ghanbari-Niaki and Rahmati-Ahmadabad, 2013; Hortemo et al., 2016; Dalise et al., 2017; Krysciak et al., 2018; Pengam et al., 2023).

Beyond physical changes, aerobic exercise also drives neuroendocrine and molecular responses. It elevates ACTH, BDNF, dopamine and serotonin levels and upregulates CREB expression, thereby supporting mental health, cognitive performance and emotional well-being (Clark et al., 2015; Lalanza et al., 2015; Arnold et al., 2020; Tunca et al., 2021). Preclinical studies show that aerobic exercise enhances hippocampal plasticity and neurogenesis, mechanisms linked to improvements in memory, mood regulation and social behaviour (Nystoriak and Bhatnagar, 2018; Li et al., 2019; Vetr et al., 2024). BDNF, in particular, emerges as a key mediator of these effects, providing a mechanistic bridge between exercise and cognitive resilience in both animal models and humans (Phillips, 2017; Mosiołek et al., 2022).

In contrast, resistance exercise exerts its strongest influence on muscular strength and endurance. While it has a smaller effect on fat reduction compared with aerobic exercise, its capacity to stimulate muscle hypertrophy is critical for preserving functional independence, particularly in older adults (Distefano and Goodpaster, 2018; Chi et al., 2020; Seo and Hwang, 2020; Eissenberg, 2022). By maintaining muscle mass and strength, resistance exercise helps sustain resting metabolic rate and enhances physical performance across the lifespan. At the molecular level, resistance exercise promotes muscle protein synthesis and modulates anabolic hormones, thereby contributing to long-term muscle health and metabolic stability (Chi et al., 2020; D'Hulst et al., 2022). Given emerging clinical and human evidence suggesting that resistance and multicomponent exercise may confer comparable or complementary cognitive benefits, future preclinical studies are warranted to systematically evaluate the neurobehavioral and mechanistic effects of resistance exercise, including potential interactions with circadian timing. Addressing this gap will be essential for advancing a comprehensive understanding of muscle–brain crosstalk and for informing precision exercise interventions in ageing and neurodegenerative populations.

Although aerobic and resistance exercise are often studied separately, both can be understood within an incorporated framework of muscle–brain crosstalk. Aerobic training primarily optimises cardiovascular and metabolic processes, while resistance training builds and maintains musculoskeletal integrity. Despite these distinct targets, both exercise types stimulate the release of signalling molecules, including BDNF, IGF-1 and neurotransmitters that connect muscle activity to brain function. In turn, the brain regulates muscle adaptation through hormonal and neural pathways. This bidirectional exchange provides a mechanistic explanation for the broad physical, cognitive, and emotional benefits of exercise (He et al., 2018; Pedersen et al., 2019).

Additionally, aerobic and resistance exercise lead to reductions in fat mass and improvements in lean muscle mass, while supporting neurobehavioral outcomes. Their mechanisms differ but are complementary: aerobic exercise maximises cardiovascular efficiency, metabolic health, and mood regulation, whereas resistance exercise is indispensable for muscle strength, independence and long-term functional capacity.

**Figure 2:**
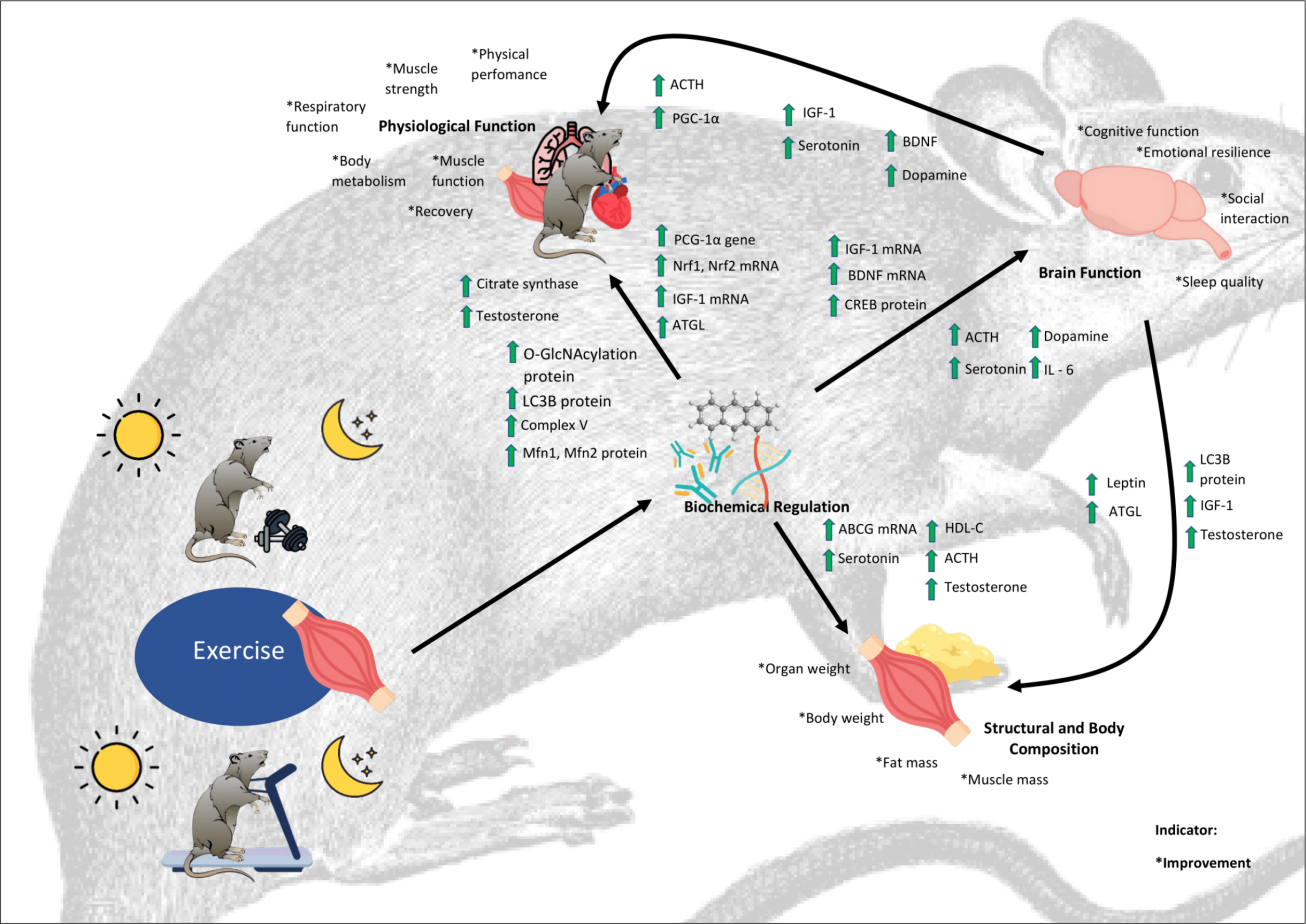
Possible crosstalk between the structural and body composition, neurobehavior, physiological and biochemical function.

**Figure 2** illustrates this integrative model by portraying the feedback loop that links exercise-induced changes in muscle and brain. This model emphasises how structural and body composition, neurobehavior, physiological and biochemical regulation responses interact dynamically through muscle–brain crosstalk, providing a systems-level perspective on how exercise supports both physical and cognitive health.

The muscle–brain crosstalk framework begins with exercise-induced changes in muscle tissue. Aerobic and resistance activities increase mitochondrial and cellular proteins, such as citrate synthase, Complex V, LC3B, and fusion proteins (Mfn1, Mfn2), which enhance energy production and muscle performance. These structural adaptations are supported by biochemical regulators, including PGC-1α, which drives mitochondrial biogenesis and hormones such as testosterone, which promote muscle growth and recovery. Together, these pathways not only strengthen muscle but also initiate signals that extend beyond the periphery (Dalise et al., 2017; Chi et al., 2020; D'Hulst et al., 2022).

These biochemical and structural shifts influence whole-body physiology. Improved metabolism and greater energy efficiency sustain prolonged activity, while endocrine signals such as IGF-1, BDNF, dopamine and serotonin directly affect the brain. These molecules support synaptic plasticity, cognition, and emotional balance, linking physical training with mental well-being (Clark et al., 2015; Lalanza et al., 2015; Arnold et al., 2020; Tunca et al., 2021). In return, brain-derived outputs, including ACTH, leptin, and neurotrophic factors modulating muscle metabolism, composition and adaptation, complete a feedback loop where muscular and neural systems regulate one another (He et al., 2018; Pedersen et al., 2019).

Mapping this loop helps explain how exercise produces multisystem benefits, but key questions remain. Most studies address short-term outcomes, leaving the long-term dynamics of muscle–brain crosstalk uncertain. Timing is another open question that circadian biology strongly influences metabolism, hormone release and cognition, yet the effects of exercise timing are still underexplored (Kim et al., 2023). In addition, aerobic and resistance training are rarely combined in trials, despite evidence that they may act synergistically (Distefano and Goodpaster, 2018; Chi et al., 2020).

Animal studies have been critical for revealing mechanisms. In rodents, exercise-induced myokines such as BDNF, IGF-1, cathepsin B and irisin promote neurogenesis, synaptic plasticity and memory (Pedersen et al., 2019). While this scoping review synthesises findings exclusively from rodent models, the insights derived can inform hypotheses for human research, particularly in areas where direct experimentation is limited or unethical. Rodents share key physiological and molecular pathways with humans (Bryda, 2013; Yarbro et al., 2025) making them suitable models for preliminary exploration. Previous studies demonstrated that humans and rodents shared the same pathway for certain diseases and physiological processes (Kurt et al., 2023; Yarbro et al., 2025). Nevertheless, differences in lifespan, brain complexity and immune responses necessitate cautious interpretation. Therefore, findings from rodent studies should be considered as foundational evidence that warrants further investigation in translational and clinical research.

Clinical evidence already shows that exercise protects against cognitive decline. Meta-analyses demonstrate that aerobic and resistance training improve memory, executive function, and hippocampal plasticity in older adults and individuals with mild cognitive impairment (Hoffmann et al., 2021; Zhang et al., 2025). In Alzheimer’s disease, aerobic and multicomponent interventions improve global cognition, with optimal benefits seen around 150 minutes of moderate activity per week (Zhang et al., 2024). What is new in the present framework is not the recognition of exercise’s cognitive benefits, but the integration of these outcomes into a mechanistic model of muscle–brain crosstalk. By linking improvements in memory and slowed cognitive decline to myokine signalling, neurotrophic factor release and circadian modulation, this model provides a biological explanation that existing scoping reviews have not fully explained.

The emerging concept of chrono-exercise adds another layer. Exercise functions as a time cue for circadian rhythms, which regulate metabolism, hormones and brain activity (Shen et al., 2023). Morning exercise may favour fat metabolism, insulin sensitivity and alertness, while evening sessions may enhance muscle growth and strength through neuromuscular readiness. Within the muscle–brain crosstalk framework, this suggests that exercise timing conditions the strength and quality of signals exchanged between muscle and brain. Although chronobiological effects are increasingly being recognised, the literature on chrono-exercise remains limited. Evidence on chrono-exercise within the included rodent studies remains limited but provides preliminary insights into time-of-day-dependent adaptations. For example, one included study demonstrated that aerobic exercise performed during the late active phase resulted in greater reductions in body weight and enhanced lipid metabolism compared with early active phase exercise (Kato et al., 2020), indicating a potential metabolic advantage associated with later exercise timing (Kato et al., 2020). Similarly, Dial et al. (2024) reported that early active phase exercise was associated with greater cardiac hypertrophy, suggesting that cardiovascular adaptations may vary depending on exercise timing (Dial et al., 2024). In contrast, resistance exercise studies showed no significant differences in muscle mass between early and late active phase training (Chi et al., 2020), indicating that timing effects may be modality-specific. Although these findings suggest that exercise timing may influence metabolic, cardiovascular, and molecular adaptations, the available evidence remains highly heterogeneous. The included studies differ substantially in exercise modality, intensity, duration, and outcome measures, and only a small number of studies directly compare different circadian phases under controlled conditions. Furthermore, neurobehavioral outcomes in relation to exercise timing were not examined in the included studies, limiting conclusions regarding cognitive and behavioural effects.

Taken together, current evidence from rodent models suggests a potential role of exercise timing in modulating physiological and biochemical responses; however, the evidence base remains insufficient to support definitive or generalizable timing recommendations. Future studies employing standardised protocols and direct phase comparisons are required to clarify the optimal timing of exercise and its mechanistic implications within the muscle–brain crosstalk framework. Given the limited and heterogeneous nature of the current evidence, it remains premature to draw definitive conclusions regarding the effects of exercise timing on physiological and behavioural outcomes.

Three research priorities stand out, (1) tailoring exercise type and timing to individual chronotype, (2) conducting long-term trials that combine aerobic and resistance training, and (3) applying omics approaches to map molecular signatures of muscle–brain signalling. These steps will determine whether mechanisms identified in animal studies apply to humans and whether precision exercise prescriptions can be developed to prevent or slow diseases such as dementia. The observed variability across studies likely reflects heterogeneity in experimental design and underlying biological context. Differences in protocol parameters, animal species and age, environmental conditions, and the timing of interventions and outcome assessments, including potential circadian influences, may substantially affect physiological and behavioural responses. Collectively, these methodological and biological factors may account for the divergent findings reported in the literature. Additionally, it is an accepted fact that the effects of exercise may differ across age groups. However, age-based stratification was outside the primary scope of this scoping review, which aimed to synthesise the overall effects of exercise interventions across the four selected fields irrespective of age. Nevertheless, the wide age range of the included studies, from young to aged rats, may have contributed to variability in physiological and behavioural responses to exercise. Accordingly, the findings should be interpreted with caution, particularly with respect to age-dependent physiological and neurobiological mechanisms. Future scoping reviews that specifically examine age-stratified outcomes are warranted to better elucidate how age modulates responses to different exercise modalities.

Although the primary focus of this scoping review is on the beneficial effects of exercise, several included and related preclinical studies provide important insights into potential adverse responses, particularly under conditions of excessive training or circadian misalignment. Evidence from rodent models indicates that high-intensity or prolonged exercise without adequate recovery may induce maladaptive physiological responses, including neuroinflammation, oxidative stress, and impaired neuroplasticity (Li et al., 2013; Ding et al., 2014; Jahangiri et al., 2019; de Souza et al., 2020). These changes are characterised by disruptions in mitochondrial function, reduced BDNF expression, and increased pro-inflammatory signalling, which may negatively affect both muscle function and cognitive outcomes.

In the preclinical rodent models’ studies, excessive exercise protocols are associated with impaired muscle–brain crosstalk, reflected by reduced BDNF expression, increased oxidative stress, and hippocampal neuronal apoptosis linked to Bax/Bcl-2 imbalance and neuroinflammatory activation (Li et al., 2013; Ding et al., 2014; Jahangiri et al., 2019). These findings suggest that the neuroprotective and metabolic benefits of exercise are load-dependent and may reverse under excessive physiological stress.

Within the context of chrono-exercise, emerging evidence further suggests that the timing of exercise may influence the threshold at which exercise shifts from adaptive to maladaptive. For instance, strenuous exercise performed during phases of heightened physiological vulnerability, such as the cortisol awakening response or late-night periods, may exacerbate stress responses, disrupt sleep architecture, and impair recovery processes (Brito et al., 2022; Kim et al., 2023). Although these findings are primarily derived from controlled preclinical models, they highlight the importance of considering both exercise dose and timing within the muscle–brain crosstalk framework.

Nevertheless, it is important to note that evidence on adverse effects remains limited and was not a primary focus of the included studies. The variability in exercise protocols, intensity thresholds, and outcome measures makes it difficult to establish clear boundaries between beneficial and detrimental adaptations. Therefore, while excessive or poorly timed exercise may pose risks, current evidence is insufficient to define precise thresholds or to generalise these findings across different populations or exercise modalities.

In summary, muscle–brain crosstalk provides an incorporated framework for understanding how exercise enhances physical, metabolic and cognitive health. Its novelty lies in bringing together mechanistic animal evidence, circadian biology and clinical outcomes into a single model. This approach moves beyond describing benefits to offering a biological rationale for personalised, time-sensitive interventions that promote health, resilience and disease prevention. This scoping review identified studies on aerobic/endurance and resistance/strength, incorporating various standard routines such as treadmill running, wheel running, swimming, jumping, and ladder climbing. These exercises were applied at different intensities and durations, with Wistar and Sprague-Dawley rats being the primary study animals. This scoping review highlights the broad and significant benefits of interventions across multiple health domains, including structural and body composition, neurobehavioral, physiological, and biochemical regulation aspects. These findings underscore the importance of diverse exercise protocols in advancing our understanding of their health benefits.

## Data Availability

The original contributions presented in the study are included in the article/supplementary material. Further inquiries can be directed to the corresponding author/s.

## References

[B1] Acevedo-Triana C. A. Rojas M. J. Cardenas F. P. (2017). Running wheel training does not change neurogenesis levels or alter working memory tasks in adult rats. PeerJ 5, e2976. doi: 10.7717/peerj.2976. PMID: 28503368 PMC5426350

[B2] Aguiar A. S. Speck A. E. Amaral I. M. Canas P. M. Cunha R. A. (2018). The exercise sex gap and the impact of the estrous cycle on exercise performance in mice. Sci. Rep. 8, 10742. doi: 10.1038/s41598-018-29050-0. PMID: 30013130 PMC6048134

[B3] Amirazodi M. Mehrabi A. Mohammad Amin R. Mohammad Abbas B. Esmaeilpour K. Daryanoosh F. . (2022). The effects of combined resveratrol and high intensity interval training on the hippocampus in aged male rats: an investigation into some signalling pathways related to mitochondria. Iran. J. Basic Med. Sci. 25, 254–262. 35655601 10.22038/IJBMS.2022.57780.12853 PMC9124540

[B4] Arksey H. O'Malley L. (2005). Scoping studies: towards a methodological framework. Int. J. Soc. Res. Method. 8, 19–32. doi: 10.1080/1364557032000119616. PMID: 39989647

[B5] Arnold M. R. Greenwood B. N. McArthur J. A. Clark P. J. Fleshner M. Lowry C. A. (2020). Effects of repeated voluntary or forced exercise on brainstem serotonergic systems in rats. Behav. Brain Res. 378. doi: 10.1016/j.bbr.2019.112237. PMID: 31525404 PMC6936333

[B6] Bartling B. Al-Robaiy S. Lehnich H. Binder L. Hiebl B. Simm A. (2017). Sex-related differences in the wheel-running activity of mice decline with increasing age. Exp. Gerontology 87, 139–147. doi: 10.1016/j.exger.2016.04.011. PMID: 27108181

[B7] Bilu C. Einat H. Zimmet P. Vishnevskia-Dai V. Schwartz W. J. Kronfeld-Schor N. (2022). Beneficial effects of voluntary wheel running on activity rhythms, metabolic state, and affect in a diurnal model of circadian disruption. Sci. Rep. 12. doi: 10.1038/s41598-022-06408-z. PMID: 35165331 PMC8844006

[B8] Botella J. Jamnick N. A. Granata C. Genders A. J. Perri E. Jabar T. . (2022). Exercise and training regulation of autophagy markers in human and rat skeletal muscle. Int. J. Mol. Sci. 23. doi: 10.3390/ijms23052619. PMID: 35269762 PMC8910616

[B9] Brito L. C. Marin T. C. Azevêdo L. Rosa-Silva J. M. Shea S. A. Thosar S. S. (2022). Chronobiology of exercise: Evaluating the best time to exercise for greater cardiovascular and metabolic benefits. Compr. Physiol. 12, 3621–3639. doi: 10.1002/j.2040-4603.2022.tb00225.x. PMID: 35766829 PMC10214902

[B10] Bryda E. C. (2013). The mighty mouse: The impact of rodents on advances in biomedical research. Mo. Med. 110, 207–211. 23829104 PMC3987984

[B11] Camandola S. Mattson M. P. (2017). Brain metabolism in health, aging, and neurodegeneration. EMBO J. 36, 1474–1492. doi: 10.15252/embj.201695810. PMID: 28438892 PMC5452017

[B12] Cao J. J. Gregoire B. R. (2023). Time of day of exercise does not affect the beneficial effect of exercise on bone structure in older female rats. Front. Physiol. 14. doi: 10.3389/fphys.2023.1142057. PMID: 37965104 PMC10641222

[B13] Casanova-Vallve N. Duglan D. Vaughan M. E. Pariollaud M. Handzlik M. K. Fan W. . (2022). Daily running enhances molecular and physiological circadian rhythms in skeletal muscle. Mol. Metab. 61. doi: 10.1016/j.molmet.2022.101504. PMID: 35470095 PMC9079800

[B14] Chi C. P. Hou C. W. Wu Y. Y. Wang T. H. Yu S. H. (2020). Night time resistance exercise alters muscular IL-6-related protein signalling, but not muscle growth after 10 weeks of resistance training in male rats. Gen. Physiol. Biophys. 39, 89–98. doi: 10.4149/gpb_2019047. PMID: 32039828

[B15] Ciorciari A. M. Irizarry E. Montaruli A. Lamia K. A. (2025). Exercise as a synchronizer: effects on circadian re-entrainment of core body temperature and metabolism following light-dark cycle inversion in mice. J. Pineal Res. 77, e70057. doi: 10.1111/jpi.70057. PMID: 40357848 PMC12070452

[B16] Clark P. J. Amat J. McConnell S. O. Ghasem P. R. Greenwood B. N. Maier S. F. . (2015). Running reduces uncontrollable stress-evoked serotonin and potentiates stress-evoked dopamine concentrations in the rat dorsal striatum. PloS One 10, e0141898. doi: 10.1371/journal.pone.0141898. PMID: 26555633 PMC4640857

[B17] Consorti A. Di Marco I. Sansevero G. (2021). Physical exercise modulates brain physiology through a network of long- and short-range cellular interactions. Front. Mol. Neurosci. 14. doi: 10.3389/fnmol.2021.710303. PMID: 34489641 PMC8417110

[B18] Curzi D. Sartini S. Guescini M. Lattanzi D. Di Palma M. Ambrogini P. . (2016). Effect of different exercise intensities on the myotendinous junction plasticity. PloS One 11, e0158059. doi: 10.1371/journal.pone.0158059. PMID: 27337061 PMC4918954

[B19] D'Hulst G. Masschelein E. De Bock K. (2022). Resistance exercise enhances long-term mTORC1 sensitivity to leucine. Mol. Metab. 66. doi: 10.1016/j.molmet.2022.101615, PMID: 36252815 PMC9626937

[B20] Dalise S. Cavalli L. Ghuman H. Wahlberg B. Gerwig M. Chisari C. . (2017). Biological effects of dosing aerobic exercise and neuromuscular electrical stimulation in rats. Sci. Rep. 7, 10830. doi: 10.1038/s41598-017-11260-7. PMID: 28883534 PMC5589775

[B21] de Souza R. F. Augusto R. L. de Moraes S. R. A. de Souza F. B. Gonçalves L. Pereira D. D. . (2020). Ultra-endurance associated with moderate exercise in rats induces cerebellar oxidative stress and impairs reactive GFAP isoform profile. Front. Mol. Neurosci. 13. doi: 10.3389/fnmol.2020.00157. PMID: 32982688 PMC7492828

[B22] Dial M. B. Malek E. M. Neblina G. A. Cooper A. R. Vaslieva N. I. Frommer R. . (2024). Effects of time-restricted exercise on activity rhythms and exercise-induced adaptations in the heart. Sci. Rep. 14. doi: 10.1038/s41598-023-50113-4. PMID: 38168503 PMC10761674

[B23] Di Liegro C. M. Schiera G. Proia P. Di Liegro I. (2019). Physical activity and brain health. Genes (Basel) 10. doi: 10.3390/genes10090720. PMID: 31533339 PMC6770965

[B24] Ding Y. Chang C. Xie L. Chen Z. Ai H. (2014). Intense exercise can cause excessive apoptosis and synapse plasticity damage in rat hippocampus through Ca²^+^ overload and endoplasmic reticulum stress-induced apoptosis pathway. Chin. Med. J. (Engl) 127, 3265–3271. doi: 10.1097/00029330-201409200-00014. PMID: 25266525

[B25] Distefano G. Goodpaster B. H. (2018). Effects of exercise and aging on skeletal muscle. Cold Spring Harb. Perspect. Med. 8. doi: 10.1101/cshperspect.a029785. PMID: 28432116 PMC5830901

[B26] Drust B. Waterhouse J. Atkinson G. Edwards B. Reilly T. (2005). Circadian rhythms in sports performance--an update. Chronobiol. Int. 22, 21–44. doi: 10.1081/cbi-200041039. PMID: 15865319

[B27] Eissenberg J. C. (2022). Working out: The molecular biology of exercise. Mo. Med. 119, 379–384. 36118818 PMC9462916

[B28] Freitas L. Bezerra A. Resende-Coelho A. Gomez-Lazaro M. Maciel L. Amorim T. . (2024). Impact of long-term swimming exercise on rat femur bone quality. Biomedicines 12, 35. doi: 10.3390/biomedicines12010035. PMID: 38255142 PMC10813774

[B29] Gabriel B. M. Zierath J. R. (2019). Circadian rhythms and exercise - re-setting the clock in metabolic disease. Nat. Rev. Endocrinol. 15, 197–206. doi: 10.1038/s41574-018-0150-x. PMID: 30655625

[B30] Gashi A. I. Gontarev S. Zivkovic V. Gjorgovski I. Azemi A. (2020). The effect of aerobic physical activity in adrenaline level in white laboratory rats. Med. Arch. 74, 84–89. doi: 10.5455/medarh.2020.74.84-89. PMID: 32577046 PMC7296409

[B31] Ghanbari-Niaki A. Rahmati-Ahmadabad S. (2013). Effects of a fixed-intensity of endurance training and pistacia atlantica supplementation on ATP-binding cassette G4 expression. Chin. Med. 8. doi: 10.1186/1749-8546-8-23. PMID: 24267473 PMC4175503

[B32] Gomez-Pinilla F. Hillman C. (2013). The influence of exercise on cognitive abilities. Compr. Physiol. 3, 403–428. doi: 10.1002/cphy.c110063. PMID: 23720292 PMC3951958

[B33] Guo W. Wong S. Li M. Liang W. Liesa M. Serra C. . (2012). Testosterone plus low-intensity physical training in late life improves functional performance, skeletal muscle mitochondrial biogenesis, and mitochondrial quality control in male mice. PloS One 7, e51180. doi: 10.1371/journal.pone.0051180. PMID: 23240002 PMC3519841

[B34] Hackney A. C. Lane A. R. (2015). Exercise and the regulation of endocrine hormones. Prog. Mol. Biol. Transl. Sci. 135, 293–311. doi: 10.1016/bs.pmbts.2015.07.001. PMID: 26477919

[B35] Hargreaves M. Spriet L. L. (2020). Skeletal muscle energy metabolism during exercise. Nat. Metab. 2, 817–828. doi: 10.1038/s42255-020-0251-4. PMID: 32747792

[B36] Hasegawa N. Fujie S. Horii N. Miyamoto-Mikami E. Tsuji K. Uchida M. . (2018). Effects of different exercise modes on arterial stiffness and nitric oxide synthesis. Med. Sci. Sports Exerc 50, 1177–1185. doi: 10.1249/mss.0000000000001567. PMID: 29381650

[B37] Hawley J. A. Hargreaves M. Joyner M. J. Zierath J. R. (2014). Integrative biology of exercise. Cell. 159, 738–749. doi: 10.1016/j.cell.2014.10.029. PMID: 25417152

[B38] He L. I. Wei W. R. Can Z. (2018). Effects of 12-week brisk walking training on exercise blood pressure in elderly patients with essential hypertension: a pilot study. Clin. Exp. hypertension (New York NY: 1993) 40, 673–679. doi: 10.1080/10641963.2018.1425416. PMID: 29363988

[B39] Hoffmann C. M. Petrov M. E. Lee R. E. (2021). Aerobic physical activity to improve memory and executive function in sedentary adults without cognitive impairment: A systematic review and meta-analysis. Prev. Med. Rep. 23, 101496. doi: 10.1016/j.pmedr.2021.101496. PMID: 34377632 PMC8327129

[B40] Hortemo K. H. Lunde P. K. Anonsen J. H. Kvaloy H. Munkvik M. Rehn T. A. . (2016). Exercise training increases protein O-GlcNAcylation in rat skeletal muscle. Physiol. Rep. 4. doi: 10.14814/phy2.12896. PMID: 27664189 PMC5037911

[B41] Hubbard E. F. Hinks A. Mashouri P. Power G. A. (2022). Influence of 4 weeks of downhill running on calcium sensitivity of rat single muscle fibres. Physiol. Rep. 10, e15450. doi: 10.14814/phy2.15450. PMID: 36222183 PMC9554763

[B42] Hüttemann M. Lee I. Malek M. H. (2012). (-)-Epicatechin maintains endurance training adaptation in mice after 14 days of detraining. FASEB J. 26, 1413–1422. doi: 10.1096/fj.11-196154, PMID: 22179525 PMC3316901

[B43] Iwayama K. Kurihara R. Nabekura Y. Kawabuchi R. Park I. Kobayashi M. . (2015). Exercise increases 24-h fat oxidation only when it is performed before breakfast. EBioMedicine 2, 2003–2009. doi: 10.1016/j.ebiom.2015.10.029. PMID: 26844280 PMC4703705

[B44] Jahangiri Z. Gholamnezhad Z. Hosseini M. Beheshti F. Kasraie N. (2019). The effects of moderate exercise and overtraining on learning and memory, hippocampal inflammatory cytokine levels, and brain oxidative stress markers in rats. J. Physiol. Sci. 69, 993–1004. doi: 10.1007/s12576-019-00719-z. PMID: 31637588 PMC10717043

[B45] Kato H. Ogasawara J. Takakura H. Shirato K. Sakurai T. Kizaki T. . (2020). Exercise training-enhanced lipolytic potency to catecholamine depends on the time of the day. Int. J. Mol. Sci. 21, 1–18. doi: 10.3390/ijms21186920. PMID: 32967199 PMC7554872

[B46] Kim N. Ka S. Park J. (2023). Effects of exercise timing and intensity on physiological circadian rhythm and sleep quality: a systematic review. Phys. Act. Nutr. 27, 52–63. doi: 10.20463/pan.2023.0029. PMID: 37946447 PMC10636512

[B47] Kim H. K. Radak Z. Takahashi M. Inami T. Shibata S. (2023). Chrono-exercise: time-of-day-dependent physiological responses to exercise. Sports Med. Health Sci. 5, 50–58. doi: 10.1016/j.smhs.2022.11.003. PMID: 36994180 PMC10040331

[B48] Krysciak K. Majerczak J. Krysciak J. Lochynski D. Kaczmarek D. Drzymala-Celichowska H. . (2018). Adaptation of motor unit contractile properties in rat medial gastrocnemius to treadmill endurance training: relationship to muscle mitochondrial biogenesis. PloS One 13, e0195704. doi: 10.1371/journal.pone.0195704, PMID: 29672614 PMC5908179

[B49] Kurt Z. Cheng J. Barrere-Cain R. McQuillen C. N. Saleem Z. Hsu N. . (2023). Shared and distinct pathways and networks genetically linked to coronary artery disease between human and mouse. Elife 12. doi: 10.7554/elife.88266. PMID: 38060277 PMC10703441

[B50] Lalanza J. F. Sanchez-Roige S. Cigarroa I. Gagliano H. Fuentes S. Armario A. . (2015). Long-term moderate treadmill exercise promotes stress-coping strategies in male and female rats. Sci. Rep. 5, 16166. doi: 10.1038/srep16166. PMID: 26538081 PMC4633642

[B51] Lee S. Hashimoto J. Suzuki T. Satoh A. (2017). The effects of exercise load during development on oxidative stress levels and antioxidant potential in adulthood. Free Radical Res. 51, 179–186. doi: 10.1080/10715762.2017.1291939. PMID: 28166650

[B52] Leise T. L. Harrington M. E. Molyneux P. C. Song I. Queenan H. Zimmerman E. . (2013). Voluntary exercise can strengthen the circadian system in aged mice. Age 35, 2137–2152. doi: 10.1007/s11357-012-9502-y. PMID: 23340916 PMC3825002

[B53] Leota J. Presby D. M. Le F. Czeisler M. Mascaro L. Capodilupo E. R. . (2025). Dose-response relationship between evening exercise and sleep. Nat. Commun. 16, 3297. doi: 10.1038/s41467-025-58271-x. PMID: 40234380 PMC12000559

[B54] Li F. Li T. Liu Y. (2016). Proteomics-based identification of the molecular signatures of liver tissues from aged rats following eight weeks of medium-intensity exercise. Oxid. Med. Cell. Longev. 2016, 3269405. doi: 10.1155/2016/3269405. PMID: 28116034 PMC5223045

[B55] Li J. Liu Y. Liu B. Li F. Hu J. Wang Q. . (2019). Mechanisms of aerobic exercise upregulating the expression of hippocampal synaptic plasticity-associated proteins in diabetic rats. Neural Plast. 2019, 7920540. doi: 10.1155/2019/7920540. PMID: 30911292 PMC6398012

[B56] Li S. Liu J. Yan H. (2013). Medium-intensity acute exhaustive exercise induces neural cell apoptosis in the rat hippocampus. Neural Regener. Res. 8, 127–132. doi: 10.3969/j.issn.1673-5374.2013.02.004, PMID: 25206482 PMC4107505

[B57] Lin Y. Jiayue Y. Zhu S. Ji S. Dai J. (2024). Swimming exercise reverses transcriptomic changes in aging mouse lens. BMC Med. Genomics 17, 1–11. doi: 10.1186/s12920-024-01839-1. PMID: 38439070 PMC10913554

[B58] Lovison K. Vieira L. Kunz R. I. Da Silva Scarton S. R. Antunes J. S. Karvat J. . (2018). Resistance exercise recovery morphology and AQP1 expression in denervated soleus muscle of Wistar rats. Motricidade 14. doi: 10.6063/motricidade.11788. PMID: 40564005

[B59] McGlory C. Devries M. C. Phillips S. M. (2017). Skeletal muscle and resistance exercise training; the role of protein synthesis in recovery and remodelling. J. Appl. Physiol. (1985) 122, 541–548. doi: 10.1152/japplphysiol.00613.2016. PMID: 27742803 PMC5401959

[B60] Mehrabi A. Nuori R. Gaeini A. Amirazodi M. Mehrtash M. Esfahlani M. A. . (2025). The antiaging and antioxidative effects of a combination of resveratrol and high-intensity interval training on the frontal lobe in aged rats: the role of SIRTS 4, SIRTS 5, SOD1, and SOD2. Oxid. Med. Cell. Longev. 2025, 8251896. doi: 10.1155/omcl/8251896. PMID: 39959582 PMC11824298

[B61] Monaco C. Whitfield J. Jain S. S. Spriet L. L. Bonen A. Holloway G. P. (2015). Activation of AMPKalpha2 is not required for mitochondrial FAT/CD36 accumulation during exercise. PloS One 10, e0126122. doi: 10.1371/journal.pone.0126122. PMID: 25965390 PMC4429092

[B62] Moshtagh P. R. Korthagen N. M. Plomp S. G. Pouran B. Castelein R. M. Zadpoor A. A. . (2018). Early signs of bone and cartilage changes induced by treadmill exercise in rats. JBMR Plus 2, 134–142. doi: 10.1002/jbm4.10029. PMID: 30283898 PMC6124204

[B63] Mosiołek A. Pietrzak M. Tabisz M. Wojtaszek W. Zabielska M. Ostrowska A. . (2022). Brain-derived neurotrophic factor (BDNF) as an indicator for effects of cognitive behavioural therapy (CBT): A systematic review. Biomedicines 11. doi: 10.3390/biomedicines11010027, PMID: 36672535 PMC9856193

[B64] Nystoriak M. A. Bhatnagar A. (2018). Cardiovascular effects and benefits of exercise. Front. Cardiovasc. Med. 5. doi: 10.3389/fcvm.2018.00135. PMID: 30324108 PMC6172294

[B65] Pedersen L. R. Olsen R. H. Anholm C. Astrup A. Eugen-Olsen J. Fenger M. . (2019). Effects of 1 year of exercise training versus combined exercise training and weight loss on body composition, low-grade inflammation and lipids in overweight patients with coronary artery disease: a randomized trial. Cardiovasc. Diabetol. 18, 127. doi: 10.1186/s12933-019-0934-x. PMID: 31575375 PMC6774219

[B66] Pengam M. Goanvec C. Moisan C. Simon B. Albacète G. Féray A. . (2023). Moderate intensity continuous versus high intensity interval training: metabolic responses of slow and fast skeletal muscles in rat. PloS One 18. doi: 10.1371/journal.pone.0292225. PMID: 37792807 PMC10550171

[B67] Phillips C. (2017). Brain-derived neurotrophic factor, depression, and physical activity: Making the neuroplastic connection. Neural Plast. 2017, 7260130. doi: 10.1155/2017/7260130. PMID: 28928987 PMC5591905

[B68] Rai M. Demontis F. (2022). Muscle-to-brain signalling via myokines and myometabolites. Brain Plast. 8, 43–63. doi: 10.3233/bpl-210133. PMID: 36448045 PMC9661353

[B69] Ramos-Filho D. Chicaybam G. de-Souza-Ferreira E. Guerra Martinez C. Kurtenbach E. Casimiro-Lopes G. . (2015). High intensity interval training (HIIT) induces specific changes in respiration and electron leakage in the mitochondria of different rat skeletal muscles. PloS One 10, e0131766. doi: 10.1371/journal.pone.0131766. PMID: 26121248 PMC4488295

[B70] Ren J. Xiao H. (2023). Exercise for mental well-being: exploring neurobiological advances and intervention effects in depression. Life. (Basel) 13. doi: 10.3390/life13071505. PMID: 37511879 PMC10381534

[B71] Sah N. Peterson B. D. Lubejko S. T. Vivar C. van Praag H. (2017). Running reorganizes the circuitry of one-week-old adult-born hippocampal neurons. Sci. Rep. 7, 10903. doi: 10.1038/s41598-017-11268-z. PMID: 28883658 PMC5589841

[B72] Sasaki H. Miyakawa H. Watanabe A. Tamura K. Shiga K. Lyu Y. . (2022). Evening rather than morning increased physical activity alters the microbiota in mice and is associated with increased body temperature and sympathetic nervous system activation. Biochim. Biophys. Acta Mol. Basis Dis. 1868. doi: 10.1016/j.bbadis.2022.166373. PMID: 35288284

[B73] Sellami M. Bragazzi N. L. Slimani M. Hayes L. Jabbour G. De Giorgio A. . (2019). The effect of exercise on glucoregulatory hormones: a countermeasure to human aging: insights from a comprehensive review of the literature. Int. J. Environ. Res. Public Health 16. doi: 10.3390/ijerph16101709. PMID: 31096708 PMC6572009

[B74] Seo D. Y. Hwang B. G. (2020). Effects of exercise training on the biochemical pathways associated with sarcopenia. Phys. Act. Nutr. 24, 32–38. doi: 10.20463/pan.2020.0019. PMID: 33108716 PMC7669465

[B75] Sheahan T. D. Copits B. A. Golden J. P. Gereau R. (2015). Voluntary exercise training: Analysis of mice in uninjured, inflammatory, and nerve-injured pain states. PloS One 10, e0133191. doi: 10.1371/journal.pone.0133191. PMID: 26196858 PMC4510282

[B76] Shen B. Ma C. Wu G. Liu H. Chen L. Yang G. (2023). Effects of exercise on circadian rhythms in humans. Front. Pharmacol. 14. doi: 10.3389/fphar.2023.1282357. PMID: 37886134 PMC10598774

[B77] Stehle J. H. Sheng Z. Hausmann L. Bechstein P. Weinmann O. Hernesniemi J. . (2021). Exercise-induced Nogo-A influences rodent motor learning in a time-dependent manner. PloS One 16. doi: 10.1371/journal.pone.0250743. PMID: 33951058 PMC8099082

[B78] Tal-Krivisky K. Kronfeld-Schor N. Einat H. (2015). Voluntary exercise enhances activity rhythms and ameliorates anxiety- and depression-like behaviours in the sand rat model of circadian rhythm-related mood changes. Physiol. Behav. 151, 441–447. doi: 10.1016/j.physbeh.2015.08.002. PMID: 26253214

[B79] Thompson R. S. Roller R. Greenwood B. N. Fleshner M. (2016). Wheel running improves REM sleep and attenuates stress-induced flattening of diurnal rhythms in F344 rats. Stress 19, 312–324. doi: 10.1080/10253890.2016.1174852. PMID: 27124542 PMC5575759

[B80] Toval A. Banos R. De la Cruz E. Morales-Delgado N. Pallares J. G. Ayad A. . (2017). Habituation training improves locomotor performance in a forced running wheel system in rats. Front. Behav. Neurosci. 11. doi: 10.3389/fnbeh.2017.00042. PMID: 28337132 PMC5340750

[B81] Tunca U. Saygin M. Ozmen O. Aslankoc R. Yalcin A. (2021). The impact of moderate-intensity swimming exercise on learning and memory in aged rats: the role of Sirtuin-1. Iran. J. Basic Med. Sci. 24, 1413–1420. 35096300 10.22038/IJBMS.2021.58145.12920 PMC8769519

[B82] van Moorsel D. Hansen J. Havekes B. Scheer F. Jorgensen J. A. Hoeks J. . (2016). Demonstration of a day-night rhythm in human skeletal muscle oxidative capacity. Mol. Metab. 5, 635–645. doi: 10.1016/j.molmet.2016.06.012. PMID: 27656401 PMC5021670

[B83] Vetr N. G. Gay N. R. Adkins J. N. Albertson B. G. Amar D. Amper M. A. S. . (2024). The impact of exercise on gene regulation in association with complex trait genetics. Nat. Commun. 15, 3346. doi: 10.1038/s41467-024-45966-w. PMID: 38693125 PMC11063075

[B84] Wen J. Fan C. Liu M. Li Q. Shi C. Wu X. . (2022). Leucine-enriched essential amino acids promote muscle protein synthesis and ameliorate exercise-induced exhaustion in prolonged endurance exercise in rats. Nutrire 47. doi: 10.1186/s41110-022-00158-8. PMID: 41872778

[B85] Yarbro J. M. Han X. Dasgupta A. Yang K. Liu D. Shrestha H. K. . (2025). Human and mouse proteomics reveals the shared pathways in Alzheimer’s disease and delayed protein turnover in the amyloidome. Nat. Commun. 16, 1533. doi: 10.1038/s41467-025-56853-3. PMID: 39934151 PMC11814087

[B86] Yasumoto Y. Nakao R. Oishi K. (2015). Free access to a running-wheel advances the phase of behavioural and physiological circadian rhythms and peripheral molecular clocks in mice. PloS One 10. doi: 10.1371/journal.pone.0116476. PMID: 25615603 PMC4304828

[B87] Yokokawa T. Kido K. Suga T. Isaka T. Hayashi T. Fujita S. (2018). Exercise-induced mitochondrial biogenesis coincides with the expression of mitochondrial translation factors in murine skeletal muscle. Physiol. Rep. 6, e13893. doi: 10.14814/phy2.13893. PMID: 30369085 PMC6204255

[B88] Zhang M. Fang W. Wang J. (2025). Effects of human concurrent aerobic and resistance training on cognitive health: A systematic review with meta-analysis. Int. J. Clin. Health Psychol. 25, 100559. doi: 10.1016/j.ijchp.2025.100559. PMID: 40226294 PMC11987655

[B89] Zhang Q. Gao Y. Wang W. Zhao X. Yu J. Huang H. (2024). Effect of resistance exercise on physical fitness, quality of life, and fatigue in patients with cancer: a systematic review. Front. Oncol. 14. doi: 10.3389/fonc.2024.1393902. PMID: 39099690 PMC11294253

